# Expression profiling identifies genes involved in neoplastic transformation of serous ovarian cancer

**DOI:** 10.1186/1471-2407-9-378

**Published:** 2009-10-23

**Authors:** Melissa A Merritt, Peter G Parsons, Tanya R Newton, Adam C Martyn, Penelope M Webb, Adèle C Green, David J Papadimos, Glen M Boyle

**Affiliations:** 1 Division of Cancer and Cell Biology, Queensland Institute of Medical Research, Brisbane, Queensland, Australia; 2 Division of Genetics and Population Health, Queensland Institute of Medical Research, Brisbane, Queensland, Australia; 3 School of Population Health, University of Queensland, Brisbane, Queensland, Australia; 4 Sullivan Nicolaides Pathology Laboratory, Brisbane, Queensland, Australia

## Abstract

**Background:**

The malignant potential of serous ovarian tumors, the most common ovarian tumor subtype, varies from benign to low malignant potential (LMP) tumors to frankly invasive cancers. Given the uncertainty about the relationship between these different forms, we compared their patterns of gene expression.

**Methods:**

Expression profiling was carried out on samples of 7 benign, 7 LMP and 28 invasive (moderate and poorly differentiated) serous tumors and four whole normal ovaries using oligonucleotide microarrays representing over 21,000 genes.

**Results:**

We identified 311 transcripts that distinguished invasive from benign tumors, and 20 transcripts that were significantly differentially expressed between invasive and LMP tumors at p < 0.01 (with multiple testing correction). Five genes that were differentially expressed between invasive and either benign or normal tissues were validated by real time PCR in an independent panel of 46 serous tumors (4 benign, 7 LMP, 35 invasive). Overexpression of *SLPI *and *WNT7A *and down-regulation of *C6orf31*, *PDGFRA *and *GLTSCR2 *were measured in invasive and LMP compared with benign and normal tissues. Over-expression of *WNT7A *in an ovarian cancer cell line led to increased migration and invasive capacity.

**Conclusion:**

These results highlight several genes that may play an important role across the spectrum of serous ovarian tumorigenesis.

## Background

In Australia, the age-standardized incidence of ovarian cancer was 11 cases per 100,000 women in 2005, and approximately 8 deaths per 100,000 women resulted from this disease in the same time period [1]. The difficulties associated with making improvements in early diagnosis of epithelial ovarian cancer partly result from a lack of knowledge regarding the pathway to tumor development. It is believed that the ovarian surface epithelium (OSE) is a common site for the initiation of ovarian carcinogenesis and most studies have identified genes involved in ovarian tumorigenesis by comparing gene expression profiles with normal OSE [2-8]. The major histological subtypes of epithelial ovarian cancer resemble neoplasms arising from other organs of the female genital tract that are derived from the Mullerian ducts during embryogenesis [9]. Thus it has been suggested that the comparison of Mullerian-appearing ovarian tumors with a tissue exhibiting mesothelial characteristics (OSE) may preferentially identify markers of Mullerian differentiation rather than true markers of neoplastic transformation [10,11].

To identify genes associated with neoplastic progression in the serous subtype of ovarian tumors, we compared gene expression in tissues that exhibited the spectrum of tumor behavior, namely benign, low malignant potential (LMP) and invasive. Compared with invasive tumors, benign tumors lack evidence of cellular atypia and are non-invasive, while LMP tumors display atypical proliferation but do not usually invade below the basement membrane of the ovarian surface epithelium [12]. Benign and LMP tumors usually result in an excellent prognosis for the patient.

In order to clarify the molecular relationships among the spectrum of serous ovarian tumors, we compared gene expression profiles of 7 LMP and 28 invasive (moderate and poorly differentiated or Grade 2 and Grade 3) serous ovarian tumors with two different reference groups: 7 serous benign ovarian tumors and four normal whole ovary specimens.

## Methods

### Tissue collections

Women aged 18-79 with suspected ovarian cancer and who were being treated at the Royal Brisbane and Women's Hospital were recruited to the study between 1999 and 2006. Chemotherapy-naïve tissues were collected during surgery and immediately frozen (for RNA extraction) or fixed in 10% buffered formalin to embed in paraffin. To ensure a homogeneous dataset in which to compare tumor behavior, analyses were restricted to tumors of the serous histological subtype and four normal whole ovary specimens collected from women having surgery for other benign gynecological conditions. Forty-two tumors (7 benign cystadenomas/cystadenofibromas, 7 LMP and 28 invasive (moderate and poorly differentiated or G2 and G3) tumors) and four normal ovarian samples were initially selected for analysis (Table 1, see Additional file 1 for detailed information on tissues analyzed by microarray analysis). A second set of tissues (4 benign, 7 LMP, 35 invasive (G2, G3) serous tumors and one normal ovary) was used for validation (Table 2, see Additional file 2 for more information on tissues analyzed by real time PCR). All tumor tissues were independently reviewed by an experienced pathologist (D.J.P.) to verify histological subtype and tumor grading and to estimate the percent epithelial content (Additional file 1). Informed consent was obtained from all participants and the study protocol was approved by the Queensland Institute of Medical Research, Royal Brisbane and Women's Hospital and the University of Queensland Human Research Ethics Review Committees. The study was conducted according to the Declaration of Helsinki Principles.

**Table 1:** Tumor grade and stage details of low malignant potential and invasive tissues analyzed by microarray hybridization

		Grade			Stage			
			
	Total	Well^1^	Mod^1^	Poor^1^	I	II	III	IV
Normal	4	-	-	-	-	-	-	-
Benign	7	-	-	-	-	-	-	-
LMP^2^	7	-	-	-	4	1	2	-
Invasive	28	-	6	22	2	4	21	1

^1 ^Well, moderate and poorly differentiated. ^2 ^Low malignant potential.

**Table 2:** Tumor grade and stage details of low malignant potential and invasive tissues analyzed by real time PCR

		Grade			Stage			
			
	Total	Well^1^	Mod^1^	Poor^1^	I	II	III	IV
Normal	1	-	-	-	-	-	-	-
Benign	4	-	-	-	-	-	-	-
LMP^2^	7	-	-	-	5	-	2	-
Invasive	35	-	5	30	-	-	29	6

^1 ^Well, moderate and poorly differentiated. ^2 ^Low malignant potential.

### RNA extraction

RNA was extracted from all tissues for oligonucleotide microarray analysis (set 1 only) and gene expression quantitation by real time PCR. Approximately 100 mg of fresh frozen tumor and normal ovary tissue was used for extraction following the protocol outlined by Newton *et al*. [13]. All RNA was treated with RNase-free DNase (Roche, Castle Hill, New South Wales, Australia) and only those samples with 28S:18S ratios ≥ 1.7 were selected to ensure high quality. RNA concentrations were estimated using the Nanodrop ND-1000 (NanoDrop Technologies Inc, Wilmington, DE).

### Expression profiling

Human Genome v 2.1 microarrays representing 21,329 genes (Qiagen Operon oligo set) were purchased from the Prostate Centre at the Vancouver General Hospital. Total RNA (20 μg) from individual tumor tissues and Universal Human Reference RNA (Stratagene, La Jolla, CA) was labeled with Cy5 and Cy3, respectively, using an amino allyl (indirect) method with the CyScribe Post-Labeling kit (Amersham Biosciences, Piscataway, NJ) as per the manufacturer's instructions. Purification of amino allyl-modified and CyDye-labeled cDNA was achieved using the CyScribe GFX Purification kit (Amersham Biosciences). For hybridization, test and reference cDNA were pooled and mixed with 20 μg Cot-1 DNA (Invitrogen, Carlsbad, CA), 20 μg of poly dA (Sigma, St. Louis, MO), 40 μg of Salmon Testis DNA (Sigma) and 60 μl DIG Easy hybridization solution (Roche). Hybridization mix was placed on the microarray slide, coverslipped and incubated overnight at 37°C in humidified hybridization chambers (TeleChem International, Sunnyvale, CA). Microarrays were washed twice in 1× SSC, 0.1% SDS for 5 min, once in 1× SSC and once in 0.1× SSC for 3 min. Microarrays were dried by centrifugation at 100 × *g *for 5 min and immediately scanned using the GMS-418 confocal scanner (Genetic MicroSystems/Affymetrix, Santa Clara, CA). Images were imported into ImaGene 5 (BioDiscovery, Marine Del Rey, CA) for data extraction. Mean pixel intensities (test to reference) in the Cy5 and Cy3 channels were imported into GeneSpring 7 for analysis (Agilent/Silicon Genetics, Redwood City, CA). All elements were reviewed manually and those of poor quality were removed from subsequent analysis. The data discussed in this publication have been deposited in the NCBI Gene Expression Omnibus (GEO, http://www.ncbi.nlm.nih.gov/geo/) and are accessible through GEO [GEO: GSE17308].

### Microarray data normalization and analysis

Fluorescent intensities were normalized to the mean expression level in each array and for each element (Lowess normalization). Data were additionally normalized to the median for each gene, or expressed relative to the median fluorescence ratios for a particular gene in all 46 samples. Gene expression data were calculated as the ratio of the test to reference fluorescent intensity. To eliminate low quality elements, those with intensities between 50 and 65,000 fluorescent units in the test (signal) channel in 75% of arrays were selected, resulting in 17,244 genes for analyses. Unsupervised hierarchical clustering methods were applied to 3,197 genes exhibiting greatest variance (≥ 0.5 SD) across all samples. Clustering trees were constructed using average linkage algorithms and Pearson's correlation. ANOVA analysis was applied to identify genes that were differentially expressed between all tumor groups and normal ovaries. Student's t-tests were applied to pair-wise comparisons between invasive and LMP or benign tumors. The Benjamini and Hochberg FDR multiple testing correction [14] was applied to all ANOVA analyses and tests were conducted at a significance level of p < 0.01. Thus, using the FDR multiple testing correction it is estimated that 1 in every 100 genes (1%) will be due to chance.

### Quantitative real time PCR

Primer sets were designed to amplify chromosome 6 open reading frame 31 (*C6orf31*; F: 5'-CACACGACTACATGCCCATC-3'; R: 5'-CGGTGAGGATGGTACAGAGC-3'), glioma tumor suppressor candidate region gene 2 (*GLTSCR2*; F: 5'-CGGTTCAAGAGCTTCCAGAG-3'; R: 5'-CTGATGGCAGCTACAACTGG-3'), platelet-derived growth factor receptor, alpha polypeptide (*PDGFRA*; F: 5'-GCGCTGACAGTGGCTACAT-3'; R: 5'-TTCAGAGGTCTGCGAGCTG-3'), secretory leukocyte protease inhibitor (*SLPI*; F: 5'-GGCTCTGGAAAGTCCTTCAAAGC-3'; R:-5' CATAAGTCACTGGGCACTT CC-3'), TCF3 (E2A) fusion partner (*TFPT*; F:-5' CGGAAGTGGAGTTTGTGTCA-3'; R: 5'-CTCGTTCACCTGCTCGATCT-3') and wingless-type MMTV integration site family member 7A (*WNT7A*) [15] for real time PCR. First strand cDNA was produced from total RNA as previously described [16]. The reaction included 5 μl of a 1/50 dilution of cDNA, 1× QuantiTect SYBR Green PCR Master Mix (Qiagen, Clifton Hill, Victoria, Australia), 0.5 μM of forward and reverse primers and 3 μl of water. PCR reactions were performed using the Corbett RotorGene 6000 (Corbett Research, Sydney, New South Wales, Australia). Gene expression levels were normalized using Beta-2-microglobulin (*B2M*) [13]. Product quantitation was determined as previously described [17]. Extreme outliers exhibiting expression levels beyond those expected were noted and removed from statistical analyses.

### Immunohistochemistry

Analysis of formalin-fixed paraffin-embedded sections from serous tumors and normal ovaries previously analyzed by microarrays was performed using antibodies directed against SLPI (NCL-SLPI 1/100 dilution; Novocastra Laboratories Ltd, Newcastle, UK)). The experimental protocol was adapted from manufacturer's instructions. Antigen retrieval was performed by autoclaving in 0.01 M trisodium citrate buffer (pH 6.0) at 105°C. Endogenous peroxidase activity was blocked with 0.5% hydrogen peroxide. Sections were blocked with 10% normal goat serum (Vector Laboratories, Burlingame, CA) and incubated overnight with primary antibody at 4°C. The DAKO Envision plus secondary antibodies (DakoCytomation, Carpinteria, CA) were applied and slides were stained using AEC+ substrate chromogen and counterstained with hematoxylin. Scoring was done by the study pathologist (D.J.P.) who was blinded to the study objectives. The percentage of cells staining and intensity of staining in both epithelial and stromal cells were recorded. Staining positivity was determined when any positive stain was noted. Negative controls (no primary antibody added) were carried out to confirm antibody specificity.

### Statistical analyses

Correlations between microarray and real time PCR experiments were assessed using Spearman's Rank Correlation. To assess differences in mRNA and protein expression of selected genes between the groups, the Kruskal Wallis test was applied to microarray and real time PCR analyses and Fisher's Exact test was used to analyze immunohistochemistry data. A *p-Value *< 0.05 was considered statistically significant.

### *WNT7A *vector construction and transfection

Full length *WNT7A *cDNA was amplified by RT-PCR from a malignant ovarian tumor using the primers 5' CGCGAATTCACTATGAACCGGAAAGCGCGGCGCTGCCTG 3' and 5' GCGACCGGTCTTGCACGTGTACATCTCCGTGCGCTC 3'. PCR products were detected and then cloned into the *Eco*RI and *Age*I sites of the pcDNA3.1/V5-His A plasmid (Invitrogen). The PCR product was verified by sequencing. OVCAR-3 cells were seeded at 2 × 10^5^/ml in 60 mm dishes and transfected the following day with 2 μg of plasmid DNA and 8 μl of Metafectine (Biontex Laboratories, Munich, Germany) as per the manufacturer's instructions. As a control, OVCAR-3 cells were transfected with the pcDNA3.1/V5-His A empty vector. Cells were selected in media containing 400 μg/ml geneticin (G-418; Invitrogen) for 3 weeks, and individual stable clones were picked for further analysis. Thereafter, clones were maintained on media containing 300 μg/ml G-418. Increased expression of WNT7A protein was confirmed by western blotting of whole cell lysates with Anti-WNT7A antibody (Q-12, Santa Cruz). Expression of beta actin served as the loading control.

### Cell growth assay

Cells were seeded in triplicate at 5,000 per microtiter well and allowed to attach. A separate plate was seeded for each time point at which cells were fixed and growth was estimated by the intensity of sulforhodamine B protein staining [18]. Experiments were repeated in triplicate and the mean ± SE values were determined in Prism 3.0 (GraphPad Software, San Diego, CA).

### Scratch wound migration and Matrigel invasion chamber system (MICS) assays

Assays were conducted as described by Pavey *et al*. [19]. Cells were seeded at 2.5 × 10^4 ^cells per well. At 0 and 24 h after introduction of the scratch wound, cells were fixed with methanol and stained with 1% crystal violet for 5 min at RT. Cells were washed with distilled water and allowed to dry before being photographed, and the width of the wound was measured. The experiment was performed on three independent occasions, with triplicate measurements each time. MICS assays were conducted using the BD BioCoat Matrigel Invasion Chamber system (BD Biosciences, Bedford, MA) as described by the manufacturer.

## Results

**Figure 1:**
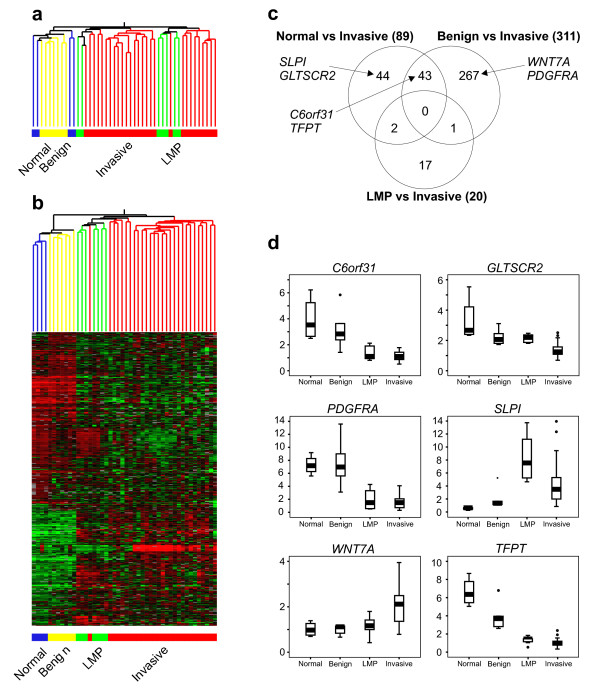
**Microarray analysis demonstrates differences in gene expression associated with tumor behavior**. Serous tumors (28 invasive, 7 low malignant potential (LMP) and 7 benign) were compared with four normal whole ovaries. (A) Unsupervised hierarchical clustering analysis of 3,197 transcripts exhibiting highest variance (SD ≥ 0.5) shows similar gene expression levels in low malignant potential and invasive tumors compared with differential expression in normal ovarian and benign tumor tissues (blue = normal ovaries; yellow = benign; green = low malignant potential; red = invasive). (B) Hierarchical clustering of 456 transcripts selected from ANOVA analysis (*p *< 0.01, Benjamini and Hochberg FDR multiple testing correction applied, Additional file 3 for genes and details) further illustrates similar gene expression profiles exhibited by low malignant potential and invasive tumors as compared with normal ovaries and benign tumors. (C) Pair-wise comparisons between invasive tumors and low malignant potential or benign tumors (*p *< 0.01, Benjamini and Hochberg FDR multiple testing correction applied) identified 20 differentially expressed genes between invasive and low malignant potential tumors and 311 genes that differed in expression between invasive and benign tumors. (D) Differential expression as detected by microarrays is demonstrated for six selected genes in serous tumors exhibiting differences in behavior. Box and whisker plots depict the median (line), interquartile range (box) and error bars demonstrate the full range of the data, excluding outliers and extreme values which are represented individually.

Unsupervised hierarchical clustering of the 3,197 transcripts with highest variance (≥ 0.5 SD) illustrated that the normal ovarian and benign tumor samples had a similar expression profile and were separate from a distinct cluster comprising all LMP and invasive tumors (Fig. 1A). Supervised analysis using ANOVA (with the Benjamini and Hochberg false discovery rate (FDR) multiple testing correction) identified 456 transcripts exhibiting significant differences in expression between the four groups (see Additional file 3 for the complete list of 456 transcripts). Hierarchical clustering to visualize the data demonstrated a similar relationship to that observed with the unsupervised analysis, with all normal ovarian and benign tumor samples in a separate cluster from all LMP and invasive tumors (Fig. 1B).

Within the group of invasive tumors, four were classified as of peritoneal rather than ovarian origin, however no distinction between gene expression profiles of ovarian and primary peritoneal tumors was observed by unsupervised clustering or ANOVA analysis (data not shown). Similarly, no differences in gene expression profiles were observed between invasive tumors of different stages (I - IV) and grades (G2 versus G3) when analyzed by unsupervised clustering or supervised ANOVA with Benjamini and Hochberg FDR multiple testing correction applied (data not shown). Further, hierarchical clustering was not affected by epithelial or tumor percentage content (data not shown).

The mean age was lowest for women with normal ovarian samples (49 years, range 41 - 57) followed by those with LMP tumors (52 years, range 25 - 76) compared with benign (61 years, range 46 - 69) and invasive tumors (62 years, range 25 - 80), although these differences did not quite reach statistical significance (*p *= 0.08, Kruskal-Wallis test). However, age had no observable effect on gene expression profiles in unsupervised clustering analysis or in supervised ANOVA analyses (with Benjamini and Hochberg FDR multiple testing correction applied) and was therefore not considered further.

The comparison of gene expression profiles of 28 invasive and seven LMP tumors identified 20 genes with expression levels that differed significantly between the two groups (*p *< 0.01, Student's t-test with Benjamini and Hochberg FDR) (gene list provided in Table 3). As expected, substantially more genes exhibiting differential expression were detected when invasive tumors were compared with seven benign tumors (311 genes) (Additional file 4 details 311 transcripts) or four normal ovaries (89 genes) (Additional file 5 lists names of the 89 transcripts). Visualization of these gene lists using a Venn diagram demonstrated very little overlap in genes that were differentially expressed between LMP and invasive tumors (20 genes) and genes that showed differential expression when invasive tumors were compared with benign tumors or normal ovaries (Fig. 1C). For example, of these 20 genes, only two were also identified in the normal *vs *invasive comparison (n = 89) and one gene was also identified in the benign *vs *invasive comparison. We performed Gene Set Enrichment Analysis (GSEA) and Expression Analysis Systematic Explorer (EASE) pathway analysis of the genes lists obtained from all of the comparisons between tumor and tissue groups. No significantly over-represented pathways were identified from either of these analyses (data not shown).

**Table 3:** Genes differentially expressed between serous invasive and low malignant potential tumors

**Genbank Acc. No**.	Gene	Description	**Fold difference**^1^	*p-*Value
NM_003514	*IGKC*	Immunoglobulin kappa constant	13.18	0.00484
NM_284764	*FTLL1*	Ferritin, light polypeptide-like 1	2.18	0.00888
NM_031157	*HNRPA1*	Heterogeneous nuclear ribonucleoprotein A1	0.69	0.00451
NM_017594	*DIRAS2*	DIRAS family, GTP-binding RAS-like 2	0.63	3.82E-05
AL122074	*SLIT3*	Slit homolog 3	0.58	0.00957
NM_003391	*WNT2*	Wingless-type MMTV integration site family member 2	0.52	0.000932
BC001356	*IFI35*	Interferon-induced protein 35	0.51	0.000297
NM_016140	*CGI-38*	Brain specific protein	0.51	0.000161
NM_078469	*BCCIP*	BRCA2 and CDKN1A interacting protein	0.5	0.00657
NM_005014	*OMD*	Osteomodulin	0.5	0.000953
AF070574	*MGC26885*	Spermatogenesis associated 2-like	0.49	0.00657
NM_013360	*ZNF222*	Zinc finger protein 222	0.47	0.00484
NM_024645	*FLJ13842*	Zinc finger, matrin type 4	0.46	0.00484
NM_004212	*SLC28A2*	Solute carrier family 28	0.45	0.00115
NM_017581	*CHRNA9*	Cholinergic receptor, nicotinic, alpha 9	0.42	0.00264
NM_006926	*SFTPA2*	Surfactant, pulmonary-associated protein A2	0.42	0.000253
AL365404	*GPR108*	G protein-coupled receptor 108	0.36	7.20E-07
NM_013941	*OR10C1*	Olfactory receptor, family 10, subfamily C, member 1	0.32	1.34E-05
NM_006934	*SLC6A9*	Solute carrier family 6, member 9	0.31	2.00E-07
NM_004720	*EDG4*	Endothelial differentiation, lysophosphatidic acid G-protein-coupled receptor, 4	0.25	3.91E-08

*P*-values were set to < 0.01 and the Benjamini and Hochberg FDR Multiple Testing Correction was applied. ^1 ^Fold difference in normalized means of invasive tumors (numerator) compared with low malignant potential tumors (denominator).

We were interested in investigating markers associated with the development of an invasive serous tumor and therefore a total of six genes were selected for further analysis, including two genes from the normal ovary *vs *invasive tumors comparison (*SLPI*, *GLTSCR2*), two genes from the benign *vs *invasive comparison (*WNT7A*, *PDGFRA*) and two genes from the overlap of these two analyses (*TFPT*, *C6orf31*) (Fig. 1C). Analyses of the microarray data for each of the six selected genes showed significant differences in expression between the groups (Kruskal Wallis *p *< 0.05, Fig. 1D).

Differences in gene expression as judged from the microarray data were compared with those from real time PCR experiments for these six genes using RNA extracted from the initial 46 tissues. Strong correlations were found between the microarray and real time PCR data for four of the candidate genes, namely *C6orf31*, *GLTSCR2*, *PDGFRA *and *SLPI *(Spearman's rank correlation coefficient ≥ 0.6, *p *< 0.001). Although similar trends in *WNT7A *expression were measured using microarray and real time PCR analyses, a poor correlation was observed (Spearman's correlation = 0.4) due to the large difference in scale of results between the two techniques. The microarray data minimized apparent differences in gene expression, possibly as they are not optimized on a single gene basis. The real time PCR data did not confirm the microarray analysis for the sixth gene (*TFPT*).

Expression levels of the five genes confirmed by real time PCR were further tested in a second independent set of tissues comprising 46 serous tumors (4 benign, 7 LMP, 35 invasive (G2/G3)) and one normal ovary. Due to the availability of only one normal ovary for validation studies, and the minimal differences in expression between normal ovarian tissue and benign lesions, we combined benign tumors with the single normal ovary for subsequent pair-wise comparisons. All candidate genes examined in the validation set exhibited similar trends in expression with tumor behavior to those observed previously in the microarray tissues. Comparing invasive tumors with grouped benign tumors and one normal ovary, all five genes demonstrated consistent and highly significant differences in gene expression (Kruskal Wallis *p *< 0.05, Table 4). Differential expression was also observed between LMP tumors and benign tumors/normal ovaries for three out of five genes examined (*PDGFRA*, *SLPI*, *WNT7A*) and between invasive and LMP tumors for the other two genes (*C6orf31*, *GLTSCR2*) (Kruskal Wallis *p *< 0.05).

**Table 4:** Validation of selected genes in an independent set of 47 tissues by real time PCR

		**Fold change (± SD)**^1^		Kruskal Wallis *p-Values*		
		
Genbank Acc. No.	Gene name	LMP^2 ^*vs *normal/benign	Invasive *vs *normal/benign	LMP^2 ^*vs *normal/benign	Invasive *vs *normal/benign	Invasive *vs *LMP^†^
NM_030651	*C6orf31*^3^	0.37 ± 0.52	0.19 ± 0.49	NS, *p *= 0.13	*p *< 0.01	*p *< 0.05
NM_015710	*GLTSCR2*^4^	0.51 ± 0.61	0.17 ± 0.22	NS, *p *= 0.17	*p *< 0.01	*p *< 0.01
NM_006206	*PDGFRA*^4^	0.31 ± 0.51	0.15 ± 0.22	*P *< 0.05	*p *< 0.01	NS, *p *= 0.13
NM_003064	*SLPI*	3.86 ± 1.80	5.70 ± 4.20	*P *< 0.05	*p *< 0.05	NS, *p *= 0.95
NM_004625	*WNT7A*	180.0 ± 250.0	150.0 ± 205.0	*P *< 0.01	*p *< 0.001	NS, *p *= 0.67

^1^Real time PCR results were calculated using the Pfaffl (2001) method with B2M as the reference gene. ^2^Low malignant potential. ^3^Outlier (00231) removed from analysis. ^4^Outliers (44286, 44428) removed from analysis.

**Figure 2:**
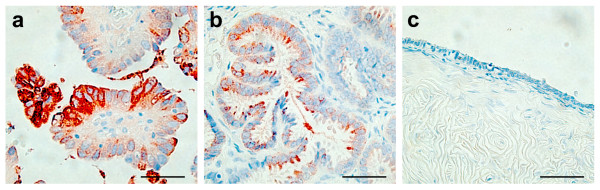
**Immunohistochemical detection of SLPI in serous ovarian tumors**. Immunohistochemistry was carried out using the SLPI antibody from Novocastra Laboratories Ltd (dilution 1/100) following the manufacturer's recommended protocol. (A) An example of an invasive serous ovarian tumor showing intense cytoplasmic staining for SLPI. (B) A low malignant potential (LMP) tumor displaying cytoplasmic staining. (C) A benign tumor with little or no staining for SLPI. Magnification: × 400. Bar = 50 μm.

To confirm differential expression at the protein level, immunohistochemistry was carried out for SLPI as a reliable, commercially-produced antibody was available. Evaluation of SLPI protein expression in the tissues used for microarray analysis showed cytoplasmic protein expression in epithelial cells in over 80% of LMP and invasive tumors compared with detectable expression in only one third of normal ovaries and benign tumors (Fisher's Exact test, *p *< 0.05 across all tissues; Table 5, Fig. 2; Additional file 6 shows representative images of tissues). SLPI staining was not observed in tumor stroma. Higher levels of gene expression measured by microarrays and real time PCR in LMP and invasive serous tumors were also correlated with increased SLPI protein expression in the paraffin-embedded tissue blocks (data not shown).

**Table 5:** Cytoplasmic expression of SLPI as detected by immunohistochemistry in 41^1 ^tissues evaluated by microarray analysis

	Normal/benign	**LMP**^2^	Invasive	All tissues	LMP *vs *Invasive
Pattern	Number (%)	Number (%)	Number (%)	*p-Value*^3^	
Negative	4 (67)	1 (14)	5 (18)	< 0.05	NS, *p-Value *= 1.0
Positive	2 (33)	6 (86)	23 (82)		

^1^Tissue blocks were not available for five samples. ^2^Low malignant potential. ^3^Fisher's Exact Test.

**Figure 3:**
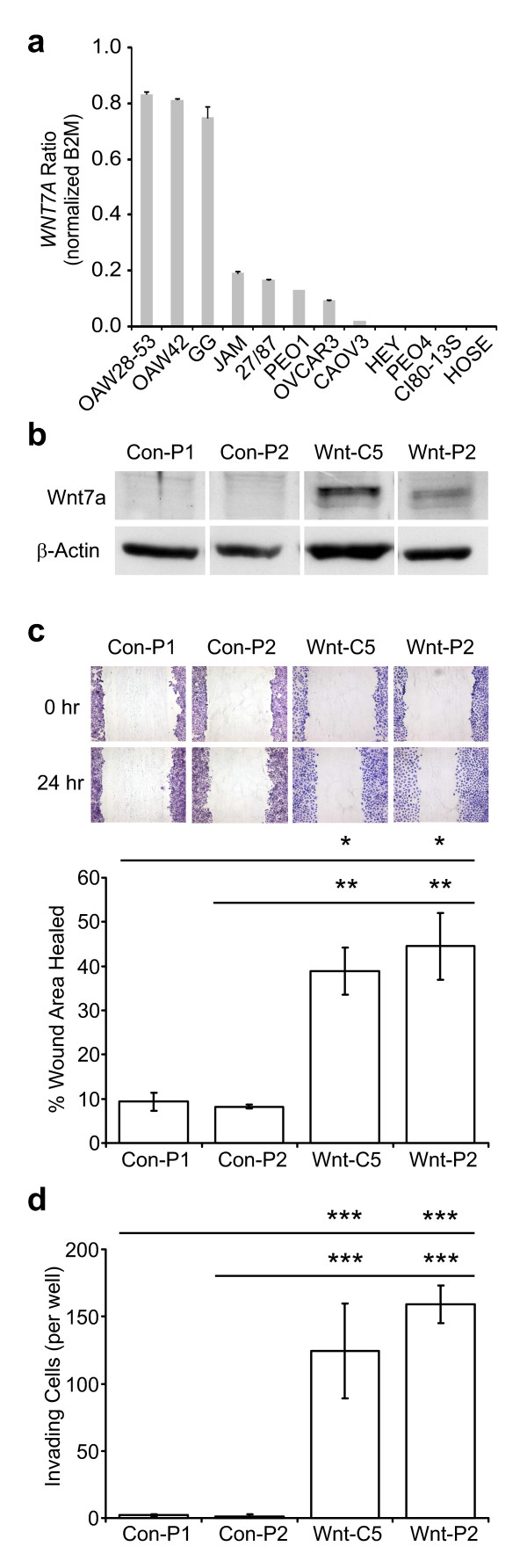
**Over-expression of *WNT7A *promotes *in vitro *migration and invasion and in OVCAR-3 ovarian cancer cells**. (A) Quantitative real-time reverse transcription-PCR detection of *WNT7A *mRNA in immortalized human ovarian surface epithelial cells (HOSE) and 11 ovarian cancer cell lines. All data was normalized to B2M expression. Data represents duplicate assays in duplicate experiments. (B) Western blot analysis confirmed higher levels of *WNT7A *in transfected clones (Wnt-C5 and Wnt-P2) as compared to the control clones (Con-P1 and Con-P2). (C) Migration ability was measured using an *in vitro *scratch wound healing assay. Above, photographs of wound regions taken immediately after or 24 hours after scratch was performed. Below, significant difference was confirmed between the *WNT7A *transfected clones and the mock clones upon measurement of the scratch wounds. (D) Invasion activity was measured *in vitro *using MICS invasion chambers; bars, SD. A dramatic increase in the number of invading cells was observed in both of the *WNT7A *transfected clones relative to the mock clones. bars, SD. *, *p *< 0.05; **, *p *< 0.02; ***, *p *< 0.01.

We wished to examine the effect of increasing expression of one of the validated genes in an ovarian cancer cell line. The largest increase observed between normal/benign and LMP/invasive tissues in validation was in *WNT7A *expression. Analysis of all WNT family members interrogated by the microarray similarly showed an increase of *WNT3 *and *WNT8A *expression in LMP and invasive tumors when compared to normal or benign tissues (Table 6; Additional file 7 shows all WNT pathway and related molecule data as taken from GSEA and EASE), possibly indicating a more widespread activation of the WNT pathway in these tumors. However, as the increase of *WNT7A *expression was the only member of the family that was statistically significant, we chose to focus on it. We therefore screened a panel of 11 ovarian cancer cell lines for expression of *WNT7A *compared with immortalized human ovarian surface epithelial cells (HOSE) (Fig. 3A). HOSE cells did not have detectable expression of *WNT7A *as determined by real time PCR, nor did 3 ovarian cancer cell lines. However, 8 of the 11 ovarian cancer cell lines showed expression of *WNT7A *(Fig. 3A). OVCAR-3, a cell line derived from ascites of a patient with adenocarcinoma of the ovary, showed low expression of *WNT7A*, and was considered a good candidate for over-expression. Far greater levels of *WNT7A *were observed in clones derived from independent transfections of the OVCAR-3 ovarian cancer cell line as compared to clones containing an empty vector, as demonstrated by both real time PCR (data not shown) and western blotting (Fig. 3B). Importantly, there was no significant difference in growth rate of the two OVCAR-3 control cell lines and the two OVCAR-3 clones expressing *WNT7A *(data not shown).

**Table 6:** Expression profiling data of WNT family members

						Ratios		
						
GenBank	Gene Name	Normal	Benign	LMP	Invasive	Invasive *vs *Normal	Invasive *vs *Benign	Invasive *vs *LMP
NM_005430	*WNT1*	1.02 ± 0.16	1.24 ± 0.22	1.03 ± 0.23	0.94 ± 0.28	0.92	0.76	0.91
NM_003391	*WNT2*	0.94 ± 0.31	1.17 ± 0.20	1.83 ± 0.44	0.94 ± 0.24	1.00	0.80	0.52*
NM_024494	*WNT2B*	1.46 ± 0.74	1.10 ± 0.68	1.03 ± 0.37	0.90 ± 0.38	0.61	0.82	0.87
NM_030753	*WNT3*	0.01 ± 0.01	0.08 ± 0.22	2.28 ± 5.64	1.32 ± 2.64	529.7	15.9	0.58
NM_033131	*WNT3A*	1.07 ± 0.21	1.20 ± 0.35	1.01 ± 0.10	0.95 ± 0.23	0.89	0.79	0.94
AB062766	*WNT4*	0.97 ± 0.13	1.10 ± 0.24	0.94 ± 0.15	0.98 ± 0.20	1.01	0.89	1.04
NM_030761	*WNT4*	1.00 ± 0.08	1.14 ± 0.16	1.10 ± 0.26	0.98 ± 0.20	0.99	0.86	0.89
NM_003392	*WNT5A*	0.91 ± 0.62	1.36 ± 0.44	1.10 ± 0.33	0.79 ± 0.35	0.87	0.58	0.72
NM_032642	*WNT5B*	1.13 ± 0.22	0.91 ± 0.16	0.96 ± 0.23	1.05 ± 0.19	0.93	1.15	1.09
NM_006522	*WNT6*	0.87 ± 0.57	1.16 ± 0.16	0.93 ± 0.11	1.05 ± 0.19	1.21	0.91	1.13
NM_004625	*WNT7A*	0.61 ± 0.19	0.60 ± 0.13	0.71 ± 0.28	1.26 ± 0.47	2.05	2.09*	1.76
NM_031933	*WNT8A*	0.52 ± 0.69	0.09 ± 0.23	2.08 ± 3.51	2.02 ± 4.72	3.85	22.9	0.97
NM_003393	*WNT8B*	1.27 ± 0.40	1.29 ± 0.33	1.55 ± 1.44	1.03 ± 0.43	0.81	0.80	0.66
NM_003395	*WNT9A*	1.14 ± 0.13	1.06 ± 0.20	1.09 ± 0.34	0.99 ± 0.31	0.87	0.94	0.91
NM_003396	*WNT9B*	1.00 ± 0.12	0.92 ± 0.49	0.99 ± 0.35	1.15 ± 0.71	1.15	1.25	1.16
NM_025216	*WNT10A*	1.23 ± 0.17	1.19 ± 0.28	1.02 ± 0.19	0.95 ± 0.20	0.78	0.80	0.93
NM_003394	*WNT10B*	0.54 ± 0.62	0.65 ± 0.63	1.04 ± 1.17	0.73 ± 0.69	1.35	1.12	0.70
NM_004626	*WNT11*	0.91 ± 0.19	1.00 ± 0.20	0.96 ± 0.10	0.97 ± 0.27	1.06	0.97	1.00
NM_057168	*WNT16*	1.01 ± 0.38	0.74 ± 0.36	1.22 ± 0.38	1.02 ± 0.41	1.01	1.39	0.84

* p < 0.01, Benjamini and Hochberg FDR multiple testing correction applied.

To determine whether *WNT7A *enhances tumorigenic potential, *in vitro *functional studies were applied to test the influence of *WNT7A *on migration and invasion capabilities in OVCAR-3 cells. Scratch wound assays showed that *WNT7A *over-expressing clones displayed a statistically significant increase in wound healing abilities as compared with control clones (Fig. 3C). *WNT7A *over-expressing clones also had significantly greater invasion ability than their mock-transfected counterparts (Fig. 3D).

## Discussion

The aim of the current study was to identify potential markers of serous ovarian neoplastic transformation. We applied gene expression profiling to evaluate the molecular relationships among serous ovarian tumors exhibiting distinct differences in tumor behavior (LMP and invasive tumors) and compared these with two reference groups, benign serous ovarian tumors and normal ovarian tissue. Unsupervised hierarchical clustering analysis demonstrated that LMP and invasive tumors exhibited similar gene expression profiles that were distinct from normal ovarian and benign tumor samples. A Student's t-test with the Benjamini and Hochberg FDR multiple testing correction applied confirmed that the gene expression profiles of invasive and LMP tumors were similar, with only 20 genes differing (at *p *< 0.01) between the two tumor types. This varied substantially from the number of genes that differed between invasive and benign tumors (311 transcripts at *p *< 0.01).

The finding of similar gene expression profiles in LMP and invasive tumors was unexpected as it is widely accepted that these tumors arise via distinct pathways [20,21]. Our results are in agreement, however, with previous findings in serous ovarian tumors [22,23] but contrast with other studies [21,24-26]. In support of the current findings of similar gene expression profiles in LMP and invasive tumors, minimal differences in gene expression profiles have been reported previously in studies that compared well-differentiated with poorly-differentiated serous invasive tumors [6,26]. Thus, the evidence currently available regarding serous ovarian cancer highlights a relatively small number of genes that may be associated with the acquisition of invasive potential. This is not unlike some corresponding findings for breast cancer where similar gene expression profiles were observed among distinct pathological stages - premalignant atypical ductal hyperplasia, pre-invasive ductal carcinoma *in situ *and invasive ductal carcinoma [27]. Further, our data may also suggest that benign ovarian lesions are a distinct entity to LMP and invasive tumors, given the differences or similarities in gene expression profile between the tissues.

We included both normal ovarian tissue and benign tumor samples for comparison with LMP and invasive serous tumors. In contrast, the majority of previous studies compared ovarian cancer (various subtypes) with the ovarian surface epithelium [2-8,24]. Contrary to our expectations, there was much overlap between differentially expressed transcripts from invasive tumor versus 'normal' tissue, regardless of whether benign tumors or normal ovaries served as the reference. Since there were significant differences between frankly malignant tumors and the above-mentioned controls, these comparisons could have minimized the differences between benign tumors and normal ovarian tissues. It is also possible that similar expression profiles in benign tumors and normal ovaries may reflect the stromal content in these samples.

From the analyses of global gene expression profiles, we focused on six genes (both novel and well-known) that were identified as differentially expressed between either normal ovarian tissue or benign tumors and invasive ovarian cancer. Five of these genes were validated as having differential expression in an independent set of serous tumors. Increased expression of SLPI was measured in LMP and invasive tumors at the mRNA and protein level. These findings support previous studies showing over-expression of SLPI in serous as well as other subtypes of ovarian cancer [28-31].

Decreased expression of *C6orf31*, *PDGFRA *and *GLTSCR2 *was measured in LMP and invasive tumors compared with high mRNA levels in benign tumors and normal ovarian tissues. To our knowledge, this is the first report of the potential involvement of *C6orf31 *(located on chromosome 6p21) in cancer development. *PDGFRA *encodes a cell surface tyrosine kinase receptor for members of the platelet-derived growth factor family. Similar observations of decreased *PDGFRA *expression in invasive serous tumors compared with benign tumors or normal ovaries have been reported in previous microarray studies [22,32]. In contrast, *PDGFRA *was also identified as overexpressed in a poor prognosis gene expression signature in ovarian cancer and it was suggested that *PDGFRA *could be associated with the occurrence of an epithelial/mesenchymal transition [33].

A trend of decreasing *GLTSCR2 *expression with increasingly aggressive tumor behavior was observed in the current study. Previous results demonstrated that down-regulation of *GLTSCR2 *directly enhanced degradation of wild-type PTEN in breast cancer (MCF7) cells [34]. Thus it was hypothesized that dysfunctional *GLTSCR2 *could lead to activation of PI3K signaling via rapid turnover of PTEN [35]. In serous ovarian cancer, amplification of multiple components of the PI3K pathway have been reported [36,37] although PTEN mutation has rarely been identified [38].

The finding of increased WNT expression in LMP and invasive tumors, in particular *WNT7A*, *WNT3 *and *WNT8A*, was of interest because it has been hypothesized that aberrant Wnt activation is associated with the initiation and growth of cancer in tissues since Wnt signaling is normally involved in growth and patterning [39]. Wnt activation, specifically *Wnt7a*, is required for the development of the female reproductive tract from the Mullerian ducts into the oviduct (fallopian tubes), uterus, cervix and upper vagina during murine embryogenesis [40,41]. *WNT7A *has previously been shown to play a role in the migration of normal cornea cells in response to wounding [42]. The function of *WNT7A *has not previously been examined in ovarian cancer. In the current study, overexpression of *WNT7A *in the ovarian cancer cell line OVCAR-3 resulted in increased cell migration and invasive capacity. The data presented here is limited by the use of a single cell line. Further analyses of additional ovarian cancer cell lines with either overexpression or ablation of *WNT7A *would be needed to precisely identify its role in ovarian cancer progression. Likewise, the role of *WNT3 *and *WNT8A *in ovarian cancer needs to be further addressed. The data presented here may suggest a widespread activation of the WNT pathway in LMP and invasive tumors, although initial pathway analysis did not indicate this to be the case. A preliminary study reported possible activation of canonical Wnt signaling in high grade serous tumors [43] but another study did not support this suggestion [44].

## Conclusion

This study showed that an unexpectedly small number of genes distinguished serous LMP and invasive tumors. Further studies of these genes may highlight those with an important role in the acquisition of invasive potential and could lead to the development of improved therapies for ovarian cancer. Molecular profiling of serous ovarian tumors exhibiting differences in behavior identified several genes potentially involved in neoplastic transformation. *In vitro *functional studies of one of these genes suggested a role for *WNT7A *in promoting migration and invasion in ovarian cancer cells. Further understanding of how serous ovarian tumors develop will contribute towards an improvement in strategies for the prevention and early detection of ovarian cancer.

## Competing interests

The authors declare that they have no competing interests.

## Authors' contributions

MAM and GMB planned and performed experiments, analyzed data and wrote the manuscript. PGP, TRN, ACM, PMW, ACG, and DJP planned experiments, analyzed data and assisted in writing the manuscript. All authors have read and approved the final manuscript.

## Pre-publication history

The pre-publication history for this paper can be accessed here:

http://www.biomedcentral.com/1471-2407/9/378/prepub

## Supplementary Material

Additional file 1 **Tissues analysed by oligonucleotide microarray**. Detailed descriptions of normal and tumor tissues analyzed by microarray analysis. Click here for file

Additional file 2 **Validation set tissues analysed by real time PCR**. Detailed descriptions of normal and tumor tissues analyzed by quantitative real time PCR analysis. Click here for file

Additional file 3 **Genes identified by ANOVA analysis of oligonucleotide microarray data**. Full gene list detailing comparisons of serous tumors (benign, LMP, invasive) *vs *normal ovaries. Click here for file

Additional file 4 **Genes differentially expressed between serous invasive and benign tumors**. Full gene list detailing comparisons of serous invasive *vs *benign tumors. Click here for file

Additional file 5 **Genes differentially expressed between serous invasive and normal ovarian tissue**. Full gene list detailing comparisons of serous invasive *vs *normal whole ovaries. Click here for file

Additional file 6 **Representative images of tissues used in the study**. H+E staining of representative tissues that were included as part of the study. Click here for file

Additional file 7 **WNT pathway and related molecules expression profiling data**. Expression profiling data of all WNT pathway and related molecules present on the microarrays. Click here for file
