# Supplementary material for: Expression profiling identifies genes involved in neoplastic transformation of serous ovarian cancer
Source: BMC Cancer. 2009 Oct 23;9:378. doi: 10.1186/1471-2407-9-378 (PMC2770078; doi:10.1186/1471-2407-9-378)
Supplement: Additional file 1 — Tissues analysed by oligonucleotide microarray. Detailed descriptions of normal and tumor tissues analyzed by microarray analysis. [file 1471-2407-9-378-S1.PDF]

Additional file 1 – Tissues analysed by oligonucleotide microarray. Data obtained from pathology reports except for grade and percent tumor which was obtained from an independent pathology review

Case	Age[1]	Primary cancer[2]	FIGO stage	Grade	Percent tumor	Specimen origin[3]
Normal						
44360	57				N/A[4]	
00157	41				N/A	
00161	48				N/A	
00192	50				N/A	
Benign						
44112	61				<1	
44121	69				5	
44155	56				<1	
44176[5]	68				5	
44324	63				10	
00096	46				5	
00252[5]	63				10	
LMP						
44047	50		1A	0	100	1°
44145	34		3B	0	100	1°
44196	76		1B	0	90	1°
44262	48		1C	0	100	1°
00007	25		3B	0	100	1°
00193	52		2A	0	90	1°
00212	76		1C	0	100	1°
Invasive						
41006	60	O	3C	2	90	2°
44033	52	O	3C	2	20	2°
44046	61	P	3C	2	50	1°
44078	56	O	3C	3	100	1°
44080	71	O	3C	3	100	NA[6]
44102	67	O	3C	2	100	2°
44110	63	O	1C	2	90	1°
44163	60	P	3C	3	30	1°
44164	66	O	3C	3	90	2°
44186	48	P/ O	3C	3	40	1°
44189	65	O	3C	3	30	1°
44234	61	P	3C	3	20	1°
44242	49	O	3C	3	95	1°
44259	77	O	4	3	60	2°
44301	73	O	3C	3	50	2°
44303	68	O	3C	3	80	2°
44306	62	O	3C	3	95	1°
44313	77	O	2C	3	40	1°
44351	64	O	3C	3	33	2°
00050	67	O	3C	3	90	2°
00056	62	O	3C	3	60	2°

Additional file 1 – Tissues analysed by oligonucletide microarray (Cont'd)

Case	Age[1]	Primary cancer[2]	FIGO stage	Grade	Percent tumor	Specimen origin[3]
Invasive (cont'd)						
00093	25	O	3C	2	100	1˚
00104	54	O	2B	3	95	1˚
00109	58	P	3C	3	100	1˚
00140	59	O	2C	3	60	1˚
00152	80	O	1C	3	100	1˚
00171	69	P	3C	3	50	1˚
00254	59	O	2C	3	90	1˚

[1] Age at time of surgery. [2] O – ovarian, P – peritoneal. [3] 1˚– diseased ovarian tissue (or omentum for primary peritoneal), 2˚ – diseased adjacent metastatic tissue. [4] Not applicable. [5] Benign section from tumor classified as LMP. [6] Not available.
