# Supplementary material for: Expression profiling identifies genes involved in neoplastic transformation of serous ovarian cancer
Source: BMC Cancer. 2009 Oct 23;9:378. doi: 10.1186/1471-2407-9-378 (PMC2770078; doi:10.1186/1471-2407-9-378)
Supplement: Additional file 2 — Validation set tissues analysed by real time PCR. Detailed descriptions of normal and tumor tissues analyzed by quantitative real time PCR analysis. [file 1471-2407-9-378-S2.PDF]

Additional file 2 – Validation set tissues analysed by real time PCR. Data obtained from pathology reports except for grade and percent tumor which was obtained from an independent pathology review

Case	Age[1]	Primary cancer[2]	FIGO stage	Grade	Percent tumor	Specimen origin
Normal						
44803	72					
Benign						
44357	75				5	
44368	42				5	
44818	68				15	
00213	64				5	
LMP						
44185	64		1A	0	30	1°
44232	66		1A	0	20	1°
44361	50		1A	0	60	1°
44400	44		3C	0	10	1°
44451	42		1C	0	40	1°
44452	23		3C	0	5	2°
44474	60		1A	0	30	1°
Invasive						
44148	76	O	4	3	50	2°
44286	63	P	4	3	5	NA[3]
44288	57	O	3C	3	40	2°
44342	76	O	3B	3	70	1°
44384	45	O	3B	3	80	1°
44409	63	O	3C	3	30	2°
44424	60	P	3C	3	20	2°
44425	73	P	4	3	70	1°
44428	69	O	3C	2	100	1°
44437	57	O	3C	3	15	2°
44446	63	P	3C	3	60	1°
44447	60	O	3C	2	95	1°
44464	69	O	3C	3	10	1°
44465	45	O	3C	3	40	2°
44469	79	O	4	3	80	1°

Additional file 2 – Validation set tissues analysed by real time PCR (Cont'd)

Case	Age[1]	Primary cancer[2]	FIGO stage	Grade	Percent tumor	Specimen origin
Invasive (cont'd)						
44472	68	P	3C	3	25	2°
44486	76	O	4	3	75	2°
44807	51	O	3C	3	15	2°
44811	71	P	3C	3	90	2°
44815	61	O	3C	3	80	1°
00010	75	O	3C	3	100	2°
00051	70	O	3C	3	80	2°
00052	63	O	3C	3	85	2°
00054	77	P	3C	3	70	1°
00063	58	O	3C	3	100	1°
00067	44	O	3C	2	100	1°
00074	76	O	3C	3	80	1°
00088	76	P	3C	3	100	1°
00103	73	O	3C	3	100	2°
00121	76	O	4	3	80	1°
00139	80	O	3C	3	90	2°
00160	62	O	3C	3	80	1°
00186	68	O	3C	3	95	2°
00194	59	O	3C	3	90	1°
00261	67	O	3C	3	80	2°

[1] Age at time of surgery. [2] O – ovarian, P – peritoneal. [3] Not available.
