# Supplementary material for: Expression profiling identifies genes involved in neoplastic transformation of serous ovarian cancer
Source: BMC Cancer. 2009 Oct 23;9:378. doi: 10.1186/1471-2407-9-378 (PMC2770078; doi:10.1186/1471-2407-9-378)
Supplement: Additional file 3 — Genes identified by ANOVA analysis of oligonucleotide microarray data. Full gene list detailing comparisons of serous tumors (benign, LMP, invasive) vs normal ovaries. [file 1471-2407-9-378-S3.PDF]

Additional file 3 – Genes identified by ANOVA analysis of oligonucleotide microarray data. Comparisons of serous tumors (benign, LMP, invasive) with normal ovaries (*p* < 0.01, Benjamini and Hochberg FDR multiple testing correction applied)

GenBank	Gene symbol	Fold difference (LMP/ ben)[1]	Fold difference (INV/ ben)[2]
NM_020682	*CYT19*	1.50	1.10
NM_004890	*SPAG7*	1.22	0.94
NM_014694	*KIAA0605*	0.68	0.73
NM_006907	*PYCR1*	0.69	0.82
AK027567	*RICH1*	0.54	0.64
NM_016056	*CGI-119*	1.90	1.93
NM_002093	*GSK3B*	1.26	1.37
NM_033540	*MFN1*	0.92	1.45
NM_015993	*TM4SF11*	1.71	1.47
NM_000436	*OXCT*	0.59	0.99
NM_017719	*SNRK*	1.40	0.93
NM_004710	*SYNGR2*	1.86	1.14
AK023938	*N/A*	0.98	1.34
NM_001908	*CTSB*	2.57	2.30
NM_000478	*ALPL*	0.57	0.59
NM_012244	*SLC7A8*	0.68	0.76
AJ242956	*N/A*	1.25	0.92
NM_021039	*S100A14*	2.93	1.95
AL031663	*C20orf171*	1.02	1.10
AK056554	*SLC2A12*	1.02	0.81
NM_004889	*ATP5J2*	1.78	2.36
NM_006082	*K-ALPHA-1*	1.18	2.17
AF353675	*FAM11A*	1.62	0.99
NM_001208	*BTF3L1*	1.31	0.83
L32832	*ATBF1*	0.52	0.48
NM_003295	*TPT1*	1.67	0.98
NM_005720	*ARPC1B*	1.33	1.64
NM_031905	*SVH*	1.63	1.99
X89067	*TRPC2*	1.18	2.44
NM_003790	*TNFRSF25*	0.51	0.53
AL136797	*FLJ20069*	1.15	0.90
NM_015956	*MRPL4*	0.79	0.72
NM_002046	*GAPD*	1.40	1.69
NM_002413	*MGST2*	1.81	1.43
AK023290	*PSMB1*	1.33	1.57
AF107405	*SFRS3*	2.59	2.91
BC017696	*MDS024*	2.64	2.90
NM_001494	*GDI2*	2.19	2.02
NM_030969	*TMEM14B*	1.30	1.69
AK022645	*N/A*	1.72	1.47
NM_004226	*STK17B*	2.18	1.86
NM_002214	*ITGB8*	4.37	3.23
NM_021238	*TERA*	3.76	3.44
AL390857	*bA51N22.1*	1.15	0.74
NM_001490	*GCNT1*	1.49	1.59
NM_031268	*PRO0461*	1.22	1.92
NM_001013	*RPS9*	1.10	0.70

GenBank	Gene symbol	Fold difference (LMP/ ben)[1]	Fold difference (INV/ ben)[2]
NM_003528	*HIST2H2BE*	0.96	1.20
BC007572	*N/A*	0.97	0.86
BC008689	*ACY-3*	1.00	0.64
AF218029	*H3F3B*	1.21	0.88
NM_021105	*PLSCR1*	1.49	1.83
NM_004058	*CAPS*	0.94	0.59
AF070628	*N/A*	1.81	1.55
AK026658	*NF1*	1.58	0.74
AB046826	*FLJ20203*	0.72	0.67
NM_014564	*LHX3*	0.74	0.73
NM_001465	*FYB*	1.71	2.00
AB058759	*KIAA1856*	0.70	0.66
NM_006135	*CAPZA1*	1.47	1.99
NM_017923	*FLJ20668*	2.63	1.43
AK054943	*HAGH*	0.59	0.58
NM_005731	*ARPC2*	1.51	1.56
NM_001450	*FHL2*	0.39	0.20
AB020642	*MYT1*	1.49	1.38
AF401222	*D4ST-1*	1.07	0.83
NM_019558	*HOXD8*	0.77	0.72
NM_006818	*AF1Q*	1.05	1.70
AL080094	*LOC286191*	0.84	0.67
NM_003750	*EIF3S10*	3.02	1.40
NM_015149	*RGL*	0.73	0.54
BC012864	*N/A*	1.71	1.55
NM_016947	*C6orf48*	0.98	0.67
D87075	*SLC23A2*	0.53	0.48
AK056791	*MUC16*	3.14	1.96
NM_000212	*ITGB3*	2.04	0.99
NM_005737	*ARL7*	2.48	2.31
AK055252	*MGC61550*	1.54	1.90
NM_057093	*CRYBA2*	1.90	1.50
BC011969	*na*	0.75	0.73
NM_004189	*SOX14*	0.57	0.60
BC017672	*MGC17791*	0.64	0.74
AK021609	*N/A*	0.71	0.78
NM_014652	*IPO13*	2.30	2.63
NM_006082	*K-ALPHA-1*	1.13	2.05
NM_014483	*RBMS3*	0.60	0.73
NM_015878	*OAZIN*	3.26	3.57
NM_002191	*INHA*	0.81	0.92
AL161420	*N/A*	2.83	1.07
M12679	*HLA-C*	1.31	2.56
NM_003794	*SNX4*	1.36	1.35
NM_006082	*K-ALPHA-1*	1.24	2.01
AK057381	*LOC90673*	1.71	1.95
NM_001953	*ECGF1*	1.43	1.34
NM_014317	*TPT*	1.21	0.90
NM_005902	*MADH3*	1.68	1.42
AK054689	*DRLM*	0.64	0.75

GenBank	Gene symbol	Fold difference (LMP/ ben)[1]	Fold difference (INV/ ben)[2]
NM_032576	*CYorf15B*	0.26	0.32
NM_001964	*EGR1*	0.35	0.47
NM_005504	*BCAT1*	2.66	1.66
NM_015414	*RPL36*	1.13	0.76
NM_002350	*LYN*	1.36	1.50
NM_005002	*NDUFA9*	2.30	2.86
NM_022359	*PDE4DIP*	0.51	0.53
NM_018970	*GPR85*	1.98	1.68
NM_021642	*FCGR2A*	2.51	3.53
NM_014271	*IL1RAPL1*	0.53	0.53
Z68274	*N/A*	1.32	0.86
NM_003919	*SGCE*	0.44	0.40
NM_015080	*NRXN2*	0.76	0.72
AI339502	*N/A*	0.56	0.67
AK055754	*N/A*	0.45	0.50
AL445468	*N/A*	0.65	0.65
AY039026	*N/A*	0.76	7.87
AK026900	*PPP1R16B*	0.54	0.68
NM_013264	*DDX25*	0.72	0.68
BC018082	*LOC124491*	0.87	0.68
NM_002127	*HLA-A*	1.16	2.12
NM_005552	*KNS2*	1.66	1.74
AK025442	*TXNDC5*	1.71	1.36
NM_012437	*SNAPAP*	0.40	0.40
NM_018975	*TERF2IP*	0.91	0.60
NM_032833	*PPP1R15B*	1.46	1.63
NM_007104	*RPL10A*	1.69	0.87
NM_006005	*WFS1*	0.49	0.51
NM_004780	*TCEAL1*	0.73	0.68
NM_024671	*FLJ23436*	0.76	0.70
NM_001660	*ARF4*	1.98	2.93
NM_002353	*TACSTD2*	3.52	3.04
AJ275401	*IGVH3*	0.74	5.47
NM_022131	*CLSTN2*	0.48	0.59
NM_006895	*HNMT*	0.60	0.55
NM_003514	*HIST1H2AM*	1.25	2.16
NM_001703	*BAI2*	0.65	0.65
NM_014585	*SLC40A1*	1.45	0.42
NM_006408	*AGR2*	2.03	0.46
AJ223812	*CALD1*	0.48	0.55
NM_016240	*SCARA3*	3.56	2.33
NM_025155	*FLJ11848*	0.53	0.62
NM_003367	*USF2*	0.72	0.68
NM_014795	*ZFHX1B*	0.51	0.62
NM_012255	*XRN2*	1.30	1.37
AL121897	*TSPYL3*	0.68	0.63
NM_013251	*TAC3*	0.80	0.74
AB018317	*KIAA0774*	0.37	0.52
AK024369	*FLJ14360*	0.88	0.75

GenBank	Gene symbol	Fold difference (LMP/ ben)[1]	Fold difference (INV/ ben)[2]
BC017730	*TNFRSF21*	2.07	1.55
AK026722	*na*	0.65	0.78
NM_024510	*MGC4368*	0.68	0.75
NM_012467	*TPSG1*	0.60	0.67
NM_005513	*GTF2E1*	0.77	0.78
NM_005442	*EOMES*	0.57	0.59
AF057036	*COLQ*	0.74	0.92
NM_018728	*MYO5C*	1.09	0.63
BC016154	*MGC16044*	0.62	0.66
NM_033332	*CDC14B*	0.77	0.72
AL137651	*DKFZp434O0213*	0.82	0.67
BC001428	*PLEKHB2*	0.88	0.78
NM_017819	*FLJ20432*	1.19	1.29
AK025277	*TNRC6*	0.69	0.73
NM_019596	*C21orf62*	0.75	0.62
BC006136	*LOC91893*	1.78	1.06
NM_015955	*CGI-27*	1.45	1.62
NM_014106	*PRO1914*	0.58	0.55
AF070574	*MGC26885*	1.88	0.92
AL161622	*N/A*	0.66	0.79
AK056251	*GDAP1L1*	0.60	0.65
NM_022450	*RHBDF1*	1.32	1.32
NM_018354	*C20orf46*	1.38	1.46
NM_001013	*RPS9*	1.21	0.71
NM_005086	*SSPN*	0.58	0.18
NM_004953	*EIF4G1*	0.94	1.22
BC016840	*MGC34695*	1.26	1.88
NM_002709	*PPP1CB*	2.19	1.98
BC012928	*C6orf150*	1.08	0.74
M11354	*H3F3A*	1.80	1.87
NM_001274	*CHEK1*	1.96	0.93
Z24725	*PLEKHC1*	0.49	0.61
NM_002164	*INDO*	1.21	1.34
BF026507	*RPEL1*	0.50	0.51
NM_023067	*FOXL2*	0.72	0.66
AK022986	*N/A*	0.64	0.66
NM_021104	*RPL41*	1.18	0.82
NM_001771	*CD22*	0.96	0.75
NM_003628	*PKP4*	1.86	2.27
AK002164	*MGC35097*	0.22	0.41
NM_016140	*CGI-38*	1.62	0.83
AJ275371	*IGVH3*	0.90	4.50
AK056789	*DKFZp434F2322*	1.08	0.76
NM_002359	*MAFG*	0.75	1.54
NM_002046	*GAPD*	1.43	1.65
NM_002577	*PAK2*	2.22	2.79
BC014198	*SEC24C*	0.76	0.64
AF181985	*JIK*	0.51	0.54
M90355	*BTF3L2*	1.22	0.74
AL049390	*N/A*	0.96	1.40
BG398014	*N/A*	0.53	5.49

GenBank	Gene symbol	Fold difference (LMP/ ben)[1]	Fold difference (INV/ ben)[2]
NM_018076	*FLJ10817*	1.34	0.71
AK022093	*N/A*	0.48	0.42
AB006627	*ASTN*	1.79	2.14
AK056446	*HSPCA*	2.50	2.11
NM_006082	*K-ALPHA-1*	1.23	2.07
NM_030674	*SLC38A1*	2.86	3.57
NM_001892	*CSNK1A1*	1.39	1.35
AL117415	*ADAM33*	0.72	0.74
M62896	*ANXA2*	2.40	1.37
X73502	*KRT20*	0.61	0.62
NM_015641	*TES*	2.24	1.63
NM_000979	*RPL18*	1.04	0.77
NM_013292	*HUMMLC2B*	0.85	0.68
NM_005500	*SAE1*	1.22	0.83
NM_002268	*KPNA4*	1.86	2.36
AK001058	*N/A*	1.85	1.87
AK024964	*NFIA*	0.71	0.63
BG535392	*IGKV1D-13*	0.76	6.13
NM_001288	*CLIC1*	1.43	1.50
U50748	*LEPR*	0.86	0.65
NM_001878	*CRABP2*	1.09	2.48
AK023815	*GALNT10*	0.99	0.93
NM_002487	*NDN*	0.54	0.56
NM_018944	*C21orf45*	0.60	0.67
NM_005005	*NDUFB9*	1.36	1.88
AJ275355	*IGVH3 V3-20*	0.75	6.56
NM_006082	*K-ALPHA-1*	1.47	2.40
NM_005526	*HSF1*	2.16	1.09
NM_004221	*NK4*	1.14	1.48
NM_002046	*GAPD*	1.50	1.81
AK023009	*DKFZp761B128*	2.51	2.61
AK055503	*na*	1.10	0.70
NM_001753	*CAV1*	0.87	0.70
NM_000444	*PHEX*	0.72	0.63
AK055745	*N/A*	0.80	0.73
AF177291	*SYNPO2*	0.19	0.22
BC001526	*MSI2*	0.84	0.66
NM_000484	*APP*	0.64	0.65
BC001255	*NCBP2*	2.04	2.51
NM_001176	*ARHGDIG*	0.75	0.78
BF541376	*N/A*	0.69	1.50
AK023024	*ALS2*	1.78	0.76
NM_017955	*CDCA4*	2.17	2.46
NM_002506	*NGFB*	1.59	1.68
NM_002730	*PRKACA*	0.73	0.69
NM_002654	*PKM2*	1.82	1.60
AL137349	*DKFZP434A0225*	5.13	4.41
NM_002178	*IGFBP6*	0.52	0.46
NM_004625	*WNT7A*	1.18	2.09
NM_003322	*TULP1*	0.63	0.65
AL049714	*RPL34P2*	1.17	0.67

GenBank	Gene symbol	Fold difference (LMP/ ben)[1]	Fold difference (INV/ ben)[2]
NM_001305	*CLDN4*	4.20	4.15
NM_000239	*LYZ*	1.99	3.01
AK000852	*LOC348262*	0.60	0.59
NM_006924	*SFRS1*	1.29	1.53
NM_001013	*RPS9*	1.24	0.75
AK057570	*FLJ33008*	0.58	0.55
NM_031297	*DKFZP761H1710*	0.53	0.56
BC009198	*na*	0.61	0.61
AL354915	*N/A*	2.37	1.69
NM_014869	*KIAA0763*	0.53	0.44
L02867	*HUMPPA*	5.50	6.22
NM_025258	*C6orf27*	0.68	0.73
AK001020	*na*	0.51	0.44
NM_005453	*ZNF297*	0.64	0.65
NM_015959	*CGI-31*	1.50	0.78
BC014245	*CTHRC1*	2.07	12.71
NM_007038	*ADAMTS5*	0.52	0.38
NM_001025	*RPS23*	1.19	0.77
NM_021999	*ITM2B*	0.79	0.54
NM_001013	*RPS9*	1.16	0.71
NM_005354	*JUND*	0.53	0.67
NM_006565	*CTCF*	3.10	2.00
NM_003588	*CUL4B*	0.75	0.71
NM_015544	*DKFZP564K1964*	0.71	0.48
NM_013230	*CD24*	3.24	3.91
AL110197	*TIMP2*	0.09	0.12
NM_001187	*BAGE*	1.34	1.63
AL137318	*N/A*	0.48	0.44
NM_014927	*CNK2*	0.42	0.38
NM_002240	*KCNJ6*	1.67	1.86
AF361486	*MUC16*	2.95	1.98
NM_024645	*FLJ13842*	1.84	0.85
NM_001145	*RNASE4*	1.26	0.82
NM_022763	*FAD104*	0.48	0.49
AJ420461	*SOXN*	0.70	0.64
NM_005313	*GRP58*	1.52	1.70
NM_006815	*RNP24*	1.78	1.77
NM_000741	*CHRM4*	0.83	0.73
NM_005014	*OMD*	1.72	0.87
NM_001955	*EDN1*	2.03	1.68
NM_002046	*GAPD*	1.44	1.68
NM_002355	*M6PR*	1.91	2.16
U17714	*ST13*	0.99	0.78
NM_000582	*SPP1*	2.19	3.50
NM_003177	*SYK*	2.17	1.29
AK058131	*FLJ25402*	0.52	0.77
NM_024822	*FLJ22843*	0.66	0.68
AK000689	*CLONE24945*	2.00	1.79
NM_006437	*ADPRTL1*	1.44	1.17
Y14737	*IGHG3*	0.74	7.24
NM_017420	*SIX4*	2.81	1.57

GenBank	Gene symbol	Fold difference (LMP/ ben)[1]	Fold difference (INV/ ben)[2]
NM_004345	*CAMP*	0.83	0.62
M90356	*BTF3L3*	1.54	0.92
NM_003891	*PROZ*	1.39	1.55
AB033073	*SULF2*	0.73	0.65
NM_003255	*TIMP2*	0.65	0.65
AJ245539	*GALNT5*	0.63	1.24
NM_003798	*CTNNAL1*	0.67	0.53
NM_002046	*GAPD*	1.54	1.82
NM_005794	*DHRS2*	0.36	0.46
NM_004343	*CALR*	1.35	2.04
NM_014624	*S100A6*	2.75	1.38
AL024493	*N/A*	1.42	1.58
NM_033103	*RHPN2*	1.54	1.36
NM_004445	*EPHB6*	0.70	0.80
NM_006206	*PDGFRA*	0.17	0.22
NM_006330	*LYPLA1*	2.87	3.92
NM_022844	*MYH11*	0.63	0.63
NM_001013	*RPS9*	1.58	0.83
NM_012200	*B3GAT3*	0.58	0.62
NM_014668	*GREB1*	0.74	0.62
NM_005929	*MFI2*	0.85	0.80
AK021816	*N/A*	0.59	0.71
NM_003768	*PEA15*	0.99	1.60
NM_004559	*NSEP1*	1.36	1.98
NM_015318	*P114-RHO-GEF*	0.57	0.55
AK055515	*D10S170*	4.97	6.04
NM_002116	*HLA-A*	0.89	2.06
NM_002046	*GAPD*	1.88	2.04
NM_005566	*LDHA*	7.05	6.83
AK054823	*OSGEP*	0.90	0.75
NM_024958	*C20orf98*	2.71	2.89
NM_002046	*GAPD*	1.62	1.98
BC017196	*CAP*	1.36	1.72
AK057676	*MGC2734*	1.85	2.42
NM_003467	*CXCR4*	1.82	5.19
NM_002046	*GAPD*	1.53	1.92
NM_004675	*ARHI*	0.32	0.29
NM_012318	*LETM1*	1.31	0.70
AB051543	*SYNE1*	1.37	0.84
NM_017581	*CHRNA9*	2.36	0.99
AK024104	*N/A*	1.17	0.78
NM_032303	*MGC10940*	0.68	0.66
NM_002937	*RNASE4*	0.77	0.72
D42044	*KIAA0090*	0.65	0.52
NM_005566	*LDHA*	6.64	6.25
NM_000581	*GPX1*	2.74	2.27
AB067506	*NaGLT1*	1.94	1.84
AK000141	*N/A*	1.73	1.36
AF116694	*CKLFSF6*	8.71	3.02
NM_004212	*SLC28A2*	2.01	0.90
NM_000504	*F10*	0.77	0.80

GenBank	Gene symbol	Fold difference (LMP/ ben)[1]	Fold difference (INV/ ben)[2]
BC014640	*COL14A1*	0.34	0.28
NM_032499	*HH114*	0.66	0.66
NM_001730	*KLF5*	4.80	2.63
AL133399	*dJ305G21.1*	3.22	2.15
BC007261	*RPL9*	1.18	0.83
NM_020547	*AMHR2*	0.70	0.61
NM_002959	*SORT1*	1.06	1.43
AK025156	*N/A*	0.91	0.69
NM_014578	*ARHD*	1.16	1.41
NM_052886	*MAL2*	4.06	4.97
NM_005023	*PGGT1B*	1.23	2.33
AF268193	*IRA1*	1.98	2.31
NM_002950	*RPN1*	2.00	2.28
AY010112	*na*	1.33	1.70
NM_031275	*TEX12*	1.31	0.68
BC012513	*ARHE*	1.89	2.07
NM_003380	*VIM*	0.72	0.42
NM_001920	*DCN*	0.19	0.31
NM_005114	*HS3ST1*	0.70	0.59
NM_005566	*LDHA*	5.65	5.76
NM_002266	*KPNA2*	1.81	4.69
NM_015515	*KRT23*	4.69	1.60
NM_005566	*LDHA*	3.62	3.60
NM_005022	*PFN1*	1.65	1.75
NM_003391	*WNT2*	1.56	0.80
NM_078469	*BCCIP*	1.45	0.73
NM_002948	*RPL15*	1.27	0.61
AF297709	*SCYL1*	0.64	0.68
AJ420521	*SMOC2*	0.44	0.38
NM_005566	*LDHA*	5.04	4.45
NM_000689	*ALDH1A1*	0.33	0.35
NM_001428	*ENO1*	1.74	2.25
NM_014464	*TINAG*	0.51	0.58
Z84469	*N/A*	1.26	0.74
NM_013360	*ZNF222*	1.58	0.74
NM_002052	*GATA4*	0.59	0.50
BC010111	*ATP5B*	2.26	2.37
NM_003064	*SLPI*	4.22	2.22
NM_005100	*AKAP12*	0.54	0.50
NM_004415	*DSP*	5.61	6.29
NM_004494	*HDGF*	1.72	2.27
NM_001152	*SLC25A5*	2.25	3.15
X77166	*SPINT3*	0.50	0.43
NM_012328	*DNAJB9*	1.88	1.90
AK056499	*PRICKLE1*	1.82	1.59
NM_006926	*SFTPA2*	2.33	0.99
X91348	*DGCR5*	0.67	0.59
NM_003926	*MBD3*	0.62	0.66
NM_000898	*MAOB*	0.55	0.45
NM_001280	*CIRBP*	1.13	0.59
NM_032298	*SYT3*	0.43	0.46

GenBank	Gene symbol	Fold difference (LMP/ ben)[1]	Fold difference (INV/ ben)[2]
AK024486	*GLTSCR2*	0.97	0.61
NM_005722	*ACTR2*	2.33	3.47
NM_005320	*HIST1H1D*	1.34	1.92
BC001356	*IFI35*	1.91	0.98
NM_024068	*MGC2731*	0.67	0.64
NM_006038	*SPATA2*	0.91	0.78
NM_002725	*PRELP*	0.57	0.52
AL122074	*SLIT3*	1.42	0.83
AL034403	*N/A*	2.74	2.96
BF664290	*na*	0.75	9.91
NM_001312	*CRIP2*	0.70	0.66
NM_052945	*TNFRSF13C*	0.72	0.67
NM_031157	*HNRPA1*	1.28	0.88
NM_017594	*DIRAS2*	1.58	1.00
AL359558	*MCC*	0.54	0.48
Z78330	*ACTR3*	1.54	1.94
AK054885	*C6orf31*	0.43	0.34
L27560	*IGFBP5*	0.20	0.28
NM_022743	*SMYD3*	1.62	1.68
NM_002387	*MCC*	0.54	0.51
NM_003348	*UBE2N*	1.66	1.71
AC003989	*WUGSC:H_RG007J15.1*	2.79	2.41
NM_005566	*LDHA*	7.14	6.07
NM_000599	*IGFBP5*	0.52	0.55
NM_005566	*LDHA*	5.14	4.69
NM_000198	*HSD3B2*	1.13	1.98
NM_001552	*IGFBP4*	0.66	0.58
AK055121	*KIAA1940*	0.54	0.51
NM_005717	*ARPC5*	2.72	4.68
NM_004234	*ZNF235*	0.63	0.36
NM_005566	*LDHA*	7.98	7.52
NM_005566	*LDHA*	9.16	9.10
NM_000224	*KRT18*	2.39	1.70
NM_014183	*DNCL2A*	0.64	0.57
NM_000349	*STAR*	0.86	0.82
M62895	*ANXA2*	3.89	2.31
AL133645	*LOC90133*	2.60	2.29
NM_013941	*OR10C1*	2.57	0.83
NM_002354	*TACSTD1*	4.13	6.88
NM_001482	*GATM*	0.47	0.31
NM_004145	*MYO9B*	0.63	0.54
NM_033014	*OGN*	0.16	0.20
NM_032760	*FLJ14009*	0.73	0.67
AL365404	*GPR108*	2.36	0.85
NM_002006	*FGF2*	0.39	0.43
AF208967	*PEG3*	0.21	0.10
NM_004126	*GNG11*	0.48	0.50
NM_006934	*SLC6A9*	2.78	0.87
AK058065	*na*	0.56	0.46
NM_004720	*EDG4*	3.03	0.77
NM_003905	*APPBP1*	0.66	0.60

GenBank	Gene symbol	Fold difference (LMP/ ben)[1]	Fold difference (INV/ ben)[2]
NM_013342	*TFPT*	0.34	0.26
NM_002932	*RMSA1*	3.02	5.42
NM_014627	*GPR57*	0.86	0.91
NM_002195	*INSL4*	0.45	0.36

[1] Fold difference in normalized means of low malignant potential (LMP) tumors (numerator) compared with benign (ben) tumors (denominator). [2] Fold difference in normalized means of invasive (INV) tumors (numerator) compared with benign (ben) tumors (denominator).
