# Supplementary material for: Expression profiling identifies genes involved in neoplastic transformation of serous ovarian cancer
Source: BMC Cancer. 2009 Oct 23;9:378. doi: 10.1186/1471-2407-9-378 (PMC2770078; doi:10.1186/1471-2407-9-378)
Supplement: Additional file 4 — Genes differentially expressed between serous invasive and benign tumors. Full gene list detailing comparisons of serous invasive vs benign tumors. [file 1471-2407-9-378-S4.PDF]

Additional file 4 – Genes differentially expressed between serous invasive and benign tumors ($p < 0.01$, Benjamini and Hochberg FDR multiple testing correction applied)

Fold difference[1]	Gene symbol	Location	GenBank	LocusLink	*p-Value*
1.75	*SDF2L1*	22q11.21	NM_022044	303116	0.00979
2.06	*TRAM1*	8q13.2	BC000687	4147	0.00979
0.70	*KIAA0676*	5q35.3	AB014576	155829	0.00979
1.52	*UBA52*	19p13.1-p12	NM_003333	5308	0.00974
1.74	*SNRPC*	6p21.31	NM_003093	1063	0.00972
0.77	*SSRP1*	11q12	NM_003146	79162	0.00971
0.69	*SLC34A3*	9	NM_017723	169758	0.00961
0.72	*LOC148066*	19p13.3	BC017592	126496	0.00961
0.75	*MGC4368*	17q25.3	NM_024510	9732	0.00961
0.67	*BTRC*	10q24-q25	NM_033637	334624	0.00953
0.64	*MGC2731*	12q13.13	NM_024068	240170	0.00953
0.48	*RNF110*	17q21.2	NM_007144	184669	0.00931
1.34	*ACTL6*	7q22	NM_016188	259831	0.00927
0.71	*ELAVL3*	19p13.2	NM_001420	1701	0.00922
2.02	*FLJ10901*	1q31.3	NM_018265	73239	0.00921
0.57	*ACVR1B*	12q13	AL117643	5288	0.00921
1.34	*ECGF1*	22q13.33	NM_001953	73946	0.00921
2.04	*CALR*	19p13.3-p13.2	NM_004343	16488	0.00921
1.58	*KIAA1949*	6p21.3	BC006176	101150	0.00921
0.69	*C14orf102*	14q32.12	NM_017970	118795	0.00919
1.50	*CLIC1*	6p22.1-p21.2	NM_001288	74276	0.00902
0.78	*GTF2E1*	3q21-q24	NM_005513	145381	0.00879
0.60	*SSTR1*	14q13	NM_001049	248160	0.00864
0.56	*PAFAH1B1*	17p13.3	NM_000430	77318	0.00841
0.65	*KIAA0404*	11q13.1	AB007864	105850	0.00839
0.55	*OGL12*	3	AF023203	158350	0.00839
1.62	*FLJ12132*	1p36.22	NM_024980	287490	0.00839
0.66	*MGC16044*	12q13.11	BC016154	350580	0.00838
0.36	*na*	2p25.2	AK057135	136753	0.00832
1.37	*GSK3B*	3q13.3	NM_002093	78802	0.00822
2.01	*K-ALPHA-1*	12q13.12	NM_006082	334842	0.00819
0.65	*N/A*		AB016195	343886	0.008
0.73	*BBC3*	19q13.3-q13.4	AF332558	87246	0.00794
0.65	*LEPR*	1p31	U50748	226627	0.00792
1.88	*MGC34695*	10q22.2	BC016840	15386	0.00777
0.72	*HOXD8*	2q31.1	NM_019558	301963	0.00776
0.29	*ARHI*	1p31	NM_004675	194695	0.00748
0.66	*RAB30*	11q12-q14	NM_014488	159505	0.0074
1.44	*FLJ20151*	15q22.1	NM_017689	279916	0.00726
1.81	*GAPD*	12p13	NM_002046	169476	0.00718
0.69	*FTL*	19q13.3-q13.4	AF119897	111334	0.00714
0.42	*VIM*	10p13	NM_003380	297753	0.00707
0.63	*PHEX*	Xp22.2-p22.1	NM_000444	72874	0.00707
2.03	*RINZF*	8q13-q21.1	NM_023929	237146	0.00696

Additional file 4 – Genes differentially expressed between serous invasive and benign tumors ($p < 0.01$, Benjamini and Hochberg FDR multiple testing correction applied) (Cont'd)

Fold difference[1]	Gene symbol	Location	GenBank	LocusLink	*p-Value*
0.57	*LOC201294*	17q25.3	AK024474	41045	0.00676
0.48	*DKFZ P564K1964*	17q12	NM_015544	3447	0.00674
2.86	*NDUFA9*	12p13.3	NM_005002	75227	0.00672
0.74	*N/A*		AK025797	288746	0.0067
1.70	*KRT18*	12q13	NM_000224	65114	0.00665
1.57	*PSMB1*	6q27	AK023290	75748	0.00664
3.57	*OAZIN*	8q22.3	NM_015878	223014	0.00661
2.09	*WNT7A*	3p25	NM_004625	72290	0.00661
0.60	*na*	8p23.1	AF131844	351404	0.00661
1.61	*MGC10999*	9q21.33	NM_032307	208914	0.00661
0.64	*SEC24C*	10q22.3	BC014198	348319	0.00661
1.95	*LOC90673*	14q11.2	AK057381	278538	0.00659
1.99	*CAPZA1*	1p13.1	NM_006135	184270	0.00656
0.63	*TSPYL3*		AL121897	302102	0.00656
0.50	*KLC2*	11q13.1	NM_022822	280792	0.00652
1.34	*DNAJB11*	3q28	NM_016306	278605	0.00652
2.63	*KLF5*	13q21.33	NM_001730	84728	0.00651
1.90	*NALP2*	19q13.42	NM_017852	6844	0.00647
1.53	*SFRS1*	17q21.3-q22	NM_006924	73737	0.00641
0.61	*PAPA-1*	2p13.1	NM_031288	118282	0.00641
0.73	*KIAA0605*	9q34.3	NM_014694	200594	0.00639
0.68	*SLC10A2*	13q33	NM_000452	194783	0.00626
0.59	*ALPL*	1p36.1-p34	NM_000478	250769	0.00626
1.45	*BEAN*	16q22.1	AK056142	100543	0.00622
1.46	*ACAA2*	18q21.1	NM_006111	32500	0.00622
0.48	*ATBF1*	16q22.3-q23.1	L32832	101842	0.00614
1.36	*N/A*		AK000141	306383	0.00614
1.87	*IMMT*	2p11.2	NM_006839	78504	0.00607
0.53	*CTNNAL1*	9q31.2	NM_003798	58488	0.00603
0.81	*CTNNB1*	3p21	NM_001904	171271	0.00596
0.56	*NDN*	15q11.2-q12	NM_002487	50130	0.00596
1.80	*SSR3*	3q25.31	NM_007107	28707	0.00595
2.09	*FLJ10074*	12q23.3	AB037781	71573	0.00595
0.64	*PBX1*	1q23	NM_002585	155691	0.00593
1.59	*GCNT1*	9q13	NM_001490	159642	0.00577
0.71	*FLJ22672*	1q22	NM_024897	235873	0.00577
0.71	*ABL1*	9q34.1	NM_005157	146355	0.00577
0.77	*CLIC3*	9q34.3	NM_004669	64746	0.00576
0.78	*PLEKHB2*	2q21.2	BC001428	307033	0.00576
0.75	*FLJ14360*	22q11.21	AK024369	351563	0.00569
1.41	*ARL6IP4*	12q24.31	NM_018694	306208	0.00548
2.63	*IPO13*	1p34.1	NM_014652	158497	0.00547
0.71	*CORO1B*	11q13.1	NM_020441	6191	0.00547
0.46	*IGFBP6*	12q13	NM_002178	274313	0.00544
2.41	*WUGSC:H RG007J15.1*	7q31	AC003989	248069	0.00539

Additional file 4 – Genes differentially expressed between serous invasive and benign tumors ($p < 0.01$, Benjamini and Hochberg FDR multiple testing correction applied) (Cont'd)

Fold difference[1]	Gene symbol	Location	GenBank	LocusLink	*p-Value*
0.48	*SLC23A2*	20p13	D87075	82042	0.00539
0.71	*S100A3*	1q21	NM_002960	2961	0.00539
1.73	*RAP2C*	Xq25	NM_021183	225979	0.00538
1.83	*PLSCR1*	3q23	NM_021105	198282	0.00538
0.65	*GDAP1L1*	20q12	AK056251	20977	0.0053
2.05	*PSMA7P*	9q22.33	AL354861	344061	0.00517
3.57	*SLC38A1*	12q13.11	NM_030674	18272	0.00517
0.55	*HNMT*	2q22.1	NM_006895	81182	0.00514
1.68	*GAPD*	12p13	NM_002046	169476	0.00513
0.72	*MRPL4*	19	NM_015956	279652	0.00511
0.71	*N/A*	N/A	AK021816	301006	0.00511
2.27	*GPX1*	3p21.3	NM_000581	76686	0.00511
0.55	*ZNF23*	16q22	AK057330	248166	0.00511
2.18	*ARPP-19*	15q21.1	AF084555	7351	0.00511
3.50	*SPP1*	4q21-q25	NM_000582	313	0.00502
2.93	*ARF4*	3p21.2-p21.1	NM_001660	75290	0.00491
0.55	*CALD1*	7q33	AJ223812	350038	0.00487
1.82	*GAPD*	12p13	NM_002046	169476	0.00483
0.62	*MUC5AC*	11p15	AJ001403	103707	0.00479
0.68	*USF2*	19q13	NM_003367	93649	0.00474
0.67	*LOC286191*	8q13.3	AL080094	41185	0.00474
0.72	*RNASE4*	14q11.1	NM_002937	283749	0.00466
0.66	*FLJ22127*	22q11.2	NM_022720	301855	0.0046
1.86	*RPS6KC1*	1q41	NM_012424	30352	0.0046
0.78	*N/A*	10p12.1	AK055602	98314	0.00457
0.51	*WFS1*	4p16	NM_006005	26077	0.00457
0.60	*SOX14*	3q22-q23	NM_004189	248184	0.00457
0.64	*LOC283687*	15q24.3	BC016975	350560	0.00442
2.29	*LOC90133*	3q26.1	AL133645	101651	0.00439
3.53	*FCGR2A*	1q23	NM_021642	78864	0.00431
0.66	*CRIP2*	14q32.3	NM_001312	70327	0.00429
6.88	*TACSTD1*	2p21	NM_002354	692	0.00427
0.68	*TCEAL1*	Xq22.1	NM_004780	95243	0.00424
2.58	*SLC3A2*	11q13	NM_002394	79748	0.00424
1.29	*FLJ20432*	3q12.3	NM_017819	57898	0.00424
1.62	*CGI-27*	2p23.2	NM_015955	20814	0.00415
1.58	*N/A*	N/A	AL024493	348528	0.00403
0.53	*IL1RAPL1*	Xp22.1-p21.3	NM_014271	241385	0.00403
0.63	*KIAA1284*	4q28.2	AB033110	27056	0.00395
0.32	*CYorf15B*	N/A	NM_032576	145010	0.00395
1.63	*PPP1R15B*	1q32.1	NM_032833	334712	0.00393
0.18	*SSPN*	12p11.2	NM_005086	183428	0.00389
2.09	*CTSC*	11q14.1-q14.3	NM_001814	10029	0.00381
1.37	*XRN2*	20p11.2-p11.1	NM_012255	268555	0.00381
1.29	*FLJ13063*	16p11.2	AK024430	121915	0.00365
2.27	*HDGF*	Xq25	NM_004494	89525	0.00353
2.11	*HSPCA*	14q32.33	AK056446	289088	0.00349

Additional file 4 – Genes differentially expressed between serous invasive and benign tumors (*p* < 0.01, Benjamini and Hochberg FDR multiple testing correction applied) (Cont'd)

Fold difference[1]	Gene symbol	Chromosome location	Genbank	LocusLink	*p-Value*
1.59	*PRICKLE1*	12q12	AK056499	6786	0.00348
2.07	*ARHE*	2q23.3	BC012513	6838	0.00348
0.65	*BAI2*	1p35	NM_001703	200586	0.00348
0.59	*EOMES*	3p21.3-p21.2	NM_005442	301704	0.00348
0.70	*FLJ23436*	16p11.2	NM_024671	85658	0.00339
0.41	*MGC35097*	3p21.31	AK002164	13781	0.00328
0.66	*N/A*	11	AK022986	38034	0.00327
2.02	*GDI2*	10p15	NM_001494	56845	0.00323
9.91	*na*	2p11.2	BF664290	247792	0.00318
2.21	*ORAOV1*	11q13.2	AK054816	62954	0.003
2.00	*FYB*	5p13.1	NM_001465	58435	0.003
0.73	*CHRM4*	11p12-p11.2	NM_000741	248100	0.003
0.52	*KIAA0090*	1p36.13	D42044	154797	0.003
0.58	*HAGH*	16p13.3	AK054943	350910	0.003
2.27	*PKP4*	2q23-q31	NM_003628	152151	0.00295
6.83	*LDHA*	11p15.4	NM_005566	2795	0.00295
1.93	*CGI-119*	12q14.1-q15	NM_016056	283670	0.00288
0.65	*TIMP2*	17q25	NM_003255	325495	0.00288
1.76	*na*	18p11.31	BC018088	351793	0.00288
3.60	*LDHA*	11p15.4	NM_005566	2795	0.00288
2.33	*PGGT1B*	5q23.1	NM_005023	766	0.00288
1.35	*SNX4*	3q21.2	NM_003794	267812	0.00288
1.87	*H3F3A*	1q41	M11354	350027	0.00288
0.67	*C21orf45*	21q22.11	NM_018944	49932	0.00288
0.68	*DDX25*	11q24	NM_013264	98738	0.00288
0.71	*C17orf31*	17p13.3	AB018275	20185	0.00288
0.68	*EDAR*	2q11-q13	NM_022336	58346	0.00288
1.44	*na*	6q23.3	AF116682	238205	0.00288
1.56	*ARPC2*	2q36.1	NM_005731	83583	0.00283
0.80	*EPHB6*	7q33-q35	NM_004445	3796	0.00275
2.79	*PAK2*	3q29	NM_002577	30692	0.00273
1.98	*GAPD*	12p13	NM_002046	169476	0.00264
1.38	*MYT1*	20q13.33	AB020642	279562	0.00261
0.40	*SNAPAP*	1q22	NM_012437	32018	0.00261
3.44	*TERA*	12p11	NM_021238	180780	0.00247
0.67	*TPSG1*	16p13.3	NM_012467	278275	0.00242
0.72	*NRXN2*	11q13	NM_015080	124085	0.00238
1.46	*C20orf46*	20p13	NM_018354	155071	0.00234
1.98	*NSEP1*	1p34	NM_004559	74497	0.00223
1.77	*RNP24*	12q24.31	NM_006815	323378	0.00209
0.75	*LOC221937*	7p22.2	AK027883	105125	0.00207
2.14	*ASTN*	1q25.2	AB006627	6788	0.00205
0.68	*HUMMLC2B*	16p12.1	NM_013292	50889	0.00205
1.68	*NGFB*	1p13.1	NM_002506	2561	0.00205
0.51	*RNF2*	1q25.2	NM_007212	124186	0.00205
0.53	*DKFZP586 D0919*	12q13.2	AL050100	49378	0.00199

Additional file 4 – Genes differentially expressed between serous invasive and benign tumors ($p < 0.01$, Benjamini and Hochberg FDR multiple testing correction applied) (Cont'd)

Fold difference[1]	Gene symbol	Location	GenBank	LocusLink	*p-Value*
2.16	*M6PR*	12p13	NM_002355	75709	0.00194
1.87	*N/A*	5	AK001058	12680	0.00194
0.66	*KIAA1883*	19q13.33	AB067470	281328	0.00193
0.65	*N/A*		AL445468	344075	0.00193
2.61	*DKFZp761B128*	12q24.31	AK023009	61976	0.00193
2.40	*K-ALPHA-1*	12q13.12	NM_006082	334842	0.00193
2.42	*MGC2734*	9q34.11	AK057676	6904	0.00193
0.43	*SPINT3; HKIB9*	20	X77166	184930	0.00191
1.44	*HERC1*	15q22	NM_003922	76127	0.00191
1.74	*KNS2*	14q32.3	NM_005552	117977	0.00189
0.55	*PRO1914*	15q13.3	NM_014106	5327	0.00189
1.68	*EDN1*	6p24.1	NM_001955	2271	0.00189
4.41	*DKFZP434A0225*	7q21.3	AL137349	83293	0.00189
1.75	*PFN1*	17p13.3	NM_005022	75721	0.00189
0.44	*na*	3q13.31	AK001020	104627	0.00187
1.92	*GAPD*	12p13	NM_002046	169476	0.00181
2.25	*ENO1*	1p36.3-p36.2	NM_001428	254105	0.00179
0.66	*MBD3*	19p13.3	NM_003926	178728	0.00179
2.04	*GAPD*	12p13	NM_002046	169476	0.00179
5.19	*CXCR4*	2q21	NM_003467	89414	0.00179
0.12	*TIMP2*	17q25	AL110197	6441	0.00179
1.70	*GRP58*	15q15	NM_005313	289101	0.00179
0.78	*ARHGDIG*	16p13.3	NM_001176	121516	0.00163
0.66	*KIAA1856*	7p22.2	AB058759	350320	0.00162
0.65	*ZNF297*	6p21.3	NM_005453	206770	0.00162
0.62	*C21orf62*	21q22.1	NM_019596	213004	0.00162
0.62	*KRT20*	17q21.2	X73502	84905	0.00162
0.56	*DKFZP761H1710*		NM_031297	19165	0.00159
1.41	*ARHD*	11q14.3	NM_014578	15114	0.00154
0.73	*C6orf27*	6p21.32	NM_025258	283875	0.00154
1.32	*RHBDF1*	16p13.3	NM_022450	57988	0.00145
0.65	*TULP1*	6p21.3	NM_003322	93537	0.00142
6.25	*LDHA*	11p15.4	NM_005566	2795	0.00141
6.04	*D10S170*	10q21	AK055515	288862	0.00139
0.63	*NFIA*	1p31.3-p31.2	AK024964	173933	0.00138
2.15	*dJ305G21.1*		AL133399	302091	0.00138
0.63	*MYH11*	16p13.13-p13.12	NM_022844	78344	0.00138
0.38	*CNK2*	Xp22.13	NM_014927	100527	0.00135
2.36	*KPNA4*	3q25.33	NM_002268	302499	0.00135
0.61	*na*	16p12.1	BC009198	348989	0.00135
0.52	*RPL5*	1p22.1	U66589	180946	0.00123
0.54	*MYO9B; MYR5*	19p13.1	NM_004145	159629	0.00123
0.51	*RPEL1*	6p23	BF026507	121591	0.00122
0.49	*FAD104*	3q26.31	NM_022763	299883	0.00122
0.54	*JIK*	12q	AF181985	12040	0.0012
0.57	*DNCL2A*	20q11.21	NM_014183	100002	0.0012

Additional file 4 – Genes differentially expressed between serous invasive and benign tumors ($p < 0.01$, Benjamini and Hochberg FDR multiple testing correction applied) (Cont'd)

Fold difference[1]	Gene symbol	Location	GenBank	LocusLink	*p-Value*
3.01	*LYZ*	12q14.3	NM_000239	234734	0.0012
0.64	*SOXN*	9q34.3	AJ420461	309165	0.00119
3.92	*LYPLA1*	8q11.23	NM_006330	12540	0.00118
6.22	*HUMPPA*	17q25.2	L02867	78358	0.00117
1.72	*CAP*		BC017196	104125	0.00117
0.44	*N/A*		AL137318	306450	0.00108
2.46	*CDCA4*	14q32.33	NM_017955	34045	0.00108
0.67	*DKFZp434O0213*	22q11.23	AL137651	272317	0.00108
1.55	*PROZ*	13q34	NM_003891	1011	0.00104
0.62	*CAMP*	3p21.3	NM_004345	51120	0.00104
0.72	*CDC14B*	9q22.33	NM_033332	22116	0.00104
0.59	*LOC348262*	17	AK000852	285165	0.00103
2.37	*ATP5B*		BC010111	25	0.00103
0.61	*AMHR2*	12q13	NM_020547	123014	0.00103
0.62	*B3GAT3*	11q12.3	NM_012200	26492	0.00103
0.53	*PDE4DIP*	1q12	NM_022359	333512	0.00103
4.69	*KPNA2*	17q23.1-q23.3	NM_002266	159557	0.000967
0.74	*ADAM33*	20p13	AL117415	173716	0.000967
0.34	*C6orf31*	6p21.31	AK054885	301920	0.000959
0.28	*COL14A1*	8q23	BC014640	36131	0.000938
2.51	*NCBP2*	3q29	BC001255	240770	0.000896
0.46	*DHRS2*	14q11.2	NM_005794	272499	0.000896
1.86	*KCNJ6*	21q22.13-q22.2	NM_002240	11173	0.000896
0.52	*PRELP*	1q32	NM_002725	76494	0.000882
2.28	*RPN1*	3q21.3-q25.2	NM_002950	2280	0.000858
0.68	*FLJ22843*	Xp11.4	NM_024822	171419	0.000856
0.55	*IGFBP5*	2q33-q36	NM_000599	107169	0.000821
1.70	*na*	2q11.2	AY010112	170538	0.000786
1.90	*DNAJB9*	7q31	NM_012328	6790	0.00077
0.67	*TNFRSF13C*	22q13.1-q13.31	NM_052945	344088	0.00077
0.58	*IGFBP4*	17q12-q21.1	NM_001552	1516	0.00077
0.65	*SULF2*	20q12-q13.2	AB033073	43857	0.00077
0.22	*PDGFRA*	4q11-q13	NM_006206	74615	0.00077
5.76	*LDHA*	11p15.4	NM_005566	2795	0.00077
4.45	*LDHA*	11p15.4	NM_005566	2795	0.00077
2.31	*ANXA2*	15q21-q22	M62895	348253	0.000709
0.66	*HH114*	15q13.3	NM_032499	48348	0.000652
0.36	*ZNF235*	19q13.2	NM_004234	298089	0.000542
1.92	*HIST1H1D*	6p21.3	NM_005320	136857	0.000542
2.75	*TD-60*	1p36.13	AB040903	284146	0.000516
2.31	*IRA1*	3q26.33	AF268193	315111	0.000507
0.71	*CUL4B*	Xq23	NM_003588	155976	0.000471
1.84	*NaGLT1*	6q22	AB067506	135033	0.000471
3.47	*ACTR2*	2p14	NM_005722	42915	0.000394
0.68	*SCYL1*	11q13	AF297709	238839	0.000394
0.55	*P114-RHO-GEF*	19p13.3	NM_015318	6150	0.000394
1.63	*BAGE*	21p11.2	NM_001187	2355	0.000394

Additional file 4 – Genes differentially expressed between serous invasive and benign tumors ($p < 0.01$, Benjamini and Hochberg FDR multiple testing correction applied) (Cont'd)

Fold difference[1]	Gene symbol	Location	GenBank	LocusLink	*p-Value*
0.44	*KIAA0763*	3p25.2	NM_014869	4764	0.000383
0.65	*APP*	21q21.3	NM_000484	177486	0.000376
1.98	*HSD3B2*	1p13.1	NM_000198	825	0.00035
0.28	*IGFBP5*	2q33-q36	L27560	180324	0.000333
0.20	*OGN*	9q22	NM_033014	109439	0.000186
2.96	*N/A*		AL034403	247900	0.000177
3.15	*SLC25A5*	Xq24-q26	NM_001152	79172	0.000165
0.50	*GATA4*	8p23.1-p22	NM_002052	243987	0.000153
0.67	*FLJ14009*	19p13.3	NM_032760	334507	0.000146
0.59	*DGCR5*	22q11	X91348	335328	0.00014
1.94	*ACTR3*	2q14.1	Z78330	10927	0.00014
0.45	*MAOB*	Xp11.4-p11.3	NM_000898	82163	0.00014
0.50	*AKAP12*	6q24-q25	NM_005100	788	0.00014
1.71	*UBE2N*	12q22	NM_003348	75355	0.00014
9.10	*LDHA*	11p15.4	NM_005566	2795	0.000138
7.52	*LDHA*	11p15.4	NM_005566	2795	6.96E-05
0.51	*KIAA1940*	2p13.2	AK055121	23158	5.96E-05
4.69	*LDHA*	11p15.4	NM_005566	2795	5.84E-05
0.38	*SMOC2*	6q27	AJ420521	22209	5.78E-05
6.07	*LDHA*	11p15.4	NM_005566	2795	5.78E-05
0.35	*ALDH1A1*	9q21.13	NM_000689	76392	3.94E-05
0.22	*SYNPO2*	4q27	AF177291	246945	3.21E-05
0.60	*APPBP1*	16q22	NM_003905	61828	3.21E-05
6.29	*DSP*	6p24	NM_004415	349499	3.21E-05
4.68	*ARPC5*	1q25.2	NM_005717	82425	2.63E-05
1.68	*SMYD3*	1q44	NM_022743	8109	2.39E-05
0.46	*na*	12q24.33	AK058065	350603	1.56E-05
0.46	*SYT3*	19q13.41	NM_032298	65848	1.53E-05
0.10	*PEG3*	19q13.4	AF208967	139033	1.38E-05
0.50	*GNG11*	7q31-q32	NM_004126	83381	1.38E-05
0.51	*MCC*	5q21-q22	NM_002387	1345	1.38E-05
0.43	*FGF2*	4q26-q27	NM_002006	284244	1.38E-05
0.26	*TFPT*	19q13	NM_013342	233765	3.87E-06
0.36	*INSL4*	9p24	NM_002195	21666	1.96E-06
5.42	*RMSA1*		NM_002932	1010	4.24E-07
0.31	*GATM*	15q15.1	NM_001482	75335	2.10E-07

[1] Fold difference in normalized means of invasive tumors (numerator) compared with benign tumors (denominator).
