# Supplementary material for: Expression profiling identifies genes involved in neoplastic transformation of serous ovarian cancer
Source: BMC Cancer. 2009 Oct 23;9:378. doi: 10.1186/1471-2407-9-378 (PMC2770078; doi:10.1186/1471-2407-9-378)
Supplement: Additional file 5 — Genes differentially expressed between serous invasive and normal ovarian tissue. Full gene list detailing comparisons of serous invasive vs normal whole ovaries. [file 1471-2407-9-378-S5.PDF]

Additional file 5 – Genes differentially expressed between serous invasive and normal ovarian tissue ($p < 0.01$, Benjamini and Hochberg FDR multiple testing correction applied)

Fold difference[1]	Gene symbol	Chromosome location	GenBank	LocusLink	*p-Value*
0.13	*GALNT10*	5q33.2	AK023815	107260	0.00938
0.62	*RNASE4*	14q11.1	NM_002937	283749	0.00904
0.72	*N/A*		BC007572	334546	0.00879
0.25	*FHL1*	Xq26	NM_001449	239069	0.00878
1.61	*INDO*	8p12-p11	NM_002164	840	0.00878
0.12	*JUND*	19p13.2	NM_005354	2780	0.00878
0.50	*N/A*		Z84469	247720	0.00878
7.29	*SLPI*	20q12	NM_003064	251754	0.00862
5.18	*LDHA*	11p15.4	NM_005566	2795	0.00846
3.76	*LYPLA1*	8q11.23	NM_006330	12540	0.00846
3.84	*LDHA*	11p15.4	NM_005566	2795	0.00795
0.23	*ARHI*	1p31	NM_004675	194695	0.00783
43.88	*CTHRC1*	8q22.3	BC014245	283713	0.00783
2.64	*RPN1*	3q21.3-q25.2	NM_002950	2280	0.00783
1.65	*UBE2N*	12q22	NM_003348	75355	0.00782
0.51	*CAV1*	7q31.1	NM_001753	74034	0.00777
5.61	*LDHA*	11p15.4	NM_005566	2795	0.00751
2.80	*FLJ10490*	19q13.33	NM_022165	293686	0.00708
2.99	*HDGF*	Xq25	NM_004494	89525	0.0069
7.18	*LDHA*	11p15.4	NM_005566	2795	0.00688
4.70	*LDHA*	11p15.4	NM_005566	2795	0.00648
3.46	*WUGSC:H_RG007J15.1*	7q31	AC003989	248069	0.00578
5.65	*C20orf98*	20p13	NM_024958	286128	0.00551
0.23	*GREB1*	2p25.1	NM_014668	193914	0.00551
0.51	*MGC10940*	9q32	NM_032303	47986	0.00543
3.01	*GALNT5*	2q24.2	AJ245539	55968	0.00524
3.39	*HTR2B*	2q36.3-q37.1	NM_000867	2507	0.00524
0.62	*CD22*	19q13.1	NM_001771	171763	0.00498
0.53	*PRKACA*	19p13.1	NM_002730	77271	0.00493
0.41	*CIRBP*	19p13.3	NM_001280	119475	0.00489
2.69	*CLONE24945*	19p13.12	AK000689	30882	0.00489
2.81	*ENO1*	1p36.3-p36.2	NM_001428	254105	0.00489
0.41	*GLTSCR2*	19q13.3	AK024486	326588	0.00489
4.58	*LDHA*	11p15.4	NM_005566	2795	0.00489
0.51	*RBMS3*	3p24-p23	NM_014483	158446	0.00489
4.03	*LDHA*	11p15.4	NM_005566	2795	0.00474
0.38	*ST13*	22q13.2	U17714	119222	0.00459
8.33	*MAL2*	8	NM_052886	76550	0.00414
2.08	*PFN1*	17p13.3	NM_005022	75721	0.00414
2.22	*SORT1*	1p21.3-p13.1	NM_002959	351872	0.00414
5.64	*LDHA*	11p15.4	NM_005566	2795	0.00367
2.11	*N/A*		AK025156	306774	0.00346
0.58	*OSGEP*	14q11.2	AK054823	337260	0.00333
0.41	*RPL9*		BC007261	157850	0.00309
0.51	*SLIT3*	5q35	AL122074	333146	0.0029
0.38	*COL14A1*	8q23	BC014640	36131	0.00282

Additional file 5 – Genes differentially expressed between serous invasive and normal ovarian tissue (*p* < 0.01, Benjamini and Hochberg FDR multiple testing correction applied) (Cont'd)

Fold difference[1]	Gene symbol	Chromosome location	GenBank	LocusLink	*p-Value*
2.39	*KRT18*	12q13	NM_000224	65114	0.00251
0.42	*IGFBP5*	2q33-q36	NM_000599	107169	0.00247
2.78	*HIST2H2BE*	1q21-q23	NM_003528	2178	0.00245
0.58	*TNFRSF13C*	22q13.1-q13.31	NM_052945	344088	0.00239
1.52	*C20orf171*		AL031663	344070	0.00201
0.56	*EDAR*	2q11-q13	NM_022336	58346	0.00169
0.37	*SPINT3; HKIB9*	20	X77166	184930	0.00169
0.19	*HS3ST1*	4p16	NM_005114	40968	0.00105
1.83	*NOLA3*	15q14-q15	NM_018648	14317	0.00105
2.00	*EIF4G1*	3q27-qter	NM_004953	211568	0.00098
0.49	*MGC2731*	12q13.13	NM_024068	240170	0.000794
4.49	*LDHA*	11p15.4	NM_005566	2795	0.000784
0.28	*ZNF235*	19q13.2	NM_004234	298089	0.000713
0.34	*PRELP*	1q32	NM_002725	76494	0.000662
0.34	*GATM*	15q15.1	NM_001482	75335	0.00065
2.78	*SPATA2*	20q13.1-q13.2	NM_006038	48513	0.00065
3.98	*TINAG*	6p11.2-p12	NM_014464	127011	0.000536
0.45	*CRIP2*	14q32.3	NM_001312	70327	0.000444
0.52	*NR2F2*	15q26	NM_021005	347991	0.000444
12.51	*LDHA*	11p15.4	NM_005566	2795	0.000385
0.44	*IGFBP4*	17q12-q21.1	NM_001552	1516	0.000292
0.60	*HNRPA1*	12q13.1	NM_031157	249495	0.00018
0.28	*C6orf31*	6p21.31	AK054885	301920	0.000155
0.53	*MFI2*	3q28-q29	NM_005929	271966	0.000121
0.38	*FGF2*	4q26-q27	NM_002006	284244	0.000119
4.67	*LOC90133*	3q26.1	AL133645	101651	0.000117
0.39	*DNCL2A*	20q11.21	NM_014183	100002	0.0001
0.59	*FLJ14009*	19p13.3	NM_032760	334507	9.44E-05
0.41	*GNG11*	7q31-q32	NM_004126	83381	8.54E-05
0.05	*PEG3*	19q13.4	AF208967	139033	7.55E-05
0.36	*na*	12q24.33	AK058065	350603	7.00E-05
0.16	*ADAMTS5*	21q21.3	NM_007038	58324	5.34E-05
3.53	*F10*	13q34	NM_000504	47913	5.34E-05
5.09	*RMSA1*		NM_002932	1010	5.34E-05
0.32	*STAR*	8p11.2	NM_000349	3132	5.34E-05
0.33	*MYO9B; MYR5*	19p13.1	NM_004145	159629	4.11E-05
0.16	*OGN*	9q22	NM_033014	109439	2.85E-05
187.42	*TACSTD1*	2p21	NM_002354	692	7.72E-06
0.22	*MCC*	5q21-q22	AL359558	284252	3.72E-06
0.43	*APPBP1*	16q22	NM_003905	61828	8.93E-07
0.15	*TFPT*	19q13	NM_013342	233765	6.35E-07
104.79	*GPR57*		NM_014627	272383	5.62E-08
0.20	*INSL4*	9p24	NM_002195	21666	8.11E-11

[1] Fold difference in normalized means of invasive tumors (numerator) compared with normal ovarian tissue (denominator).
