# Supplementary material for: Expression profiling identifies genes involved in neoplastic transformation of serous ovarian cancer
Source: BMC Cancer. 2009 Oct 23;9:378. doi: 10.1186/1471-2407-9-378 (PMC2770078; doi:10.1186/1471-2407-9-378)
Supplement: Additional file 6 — Representative images of tissues used in the study. H+E staining of representative tissues that were included as part of the study. [file 1471-2407-9-378-S6.PDF]

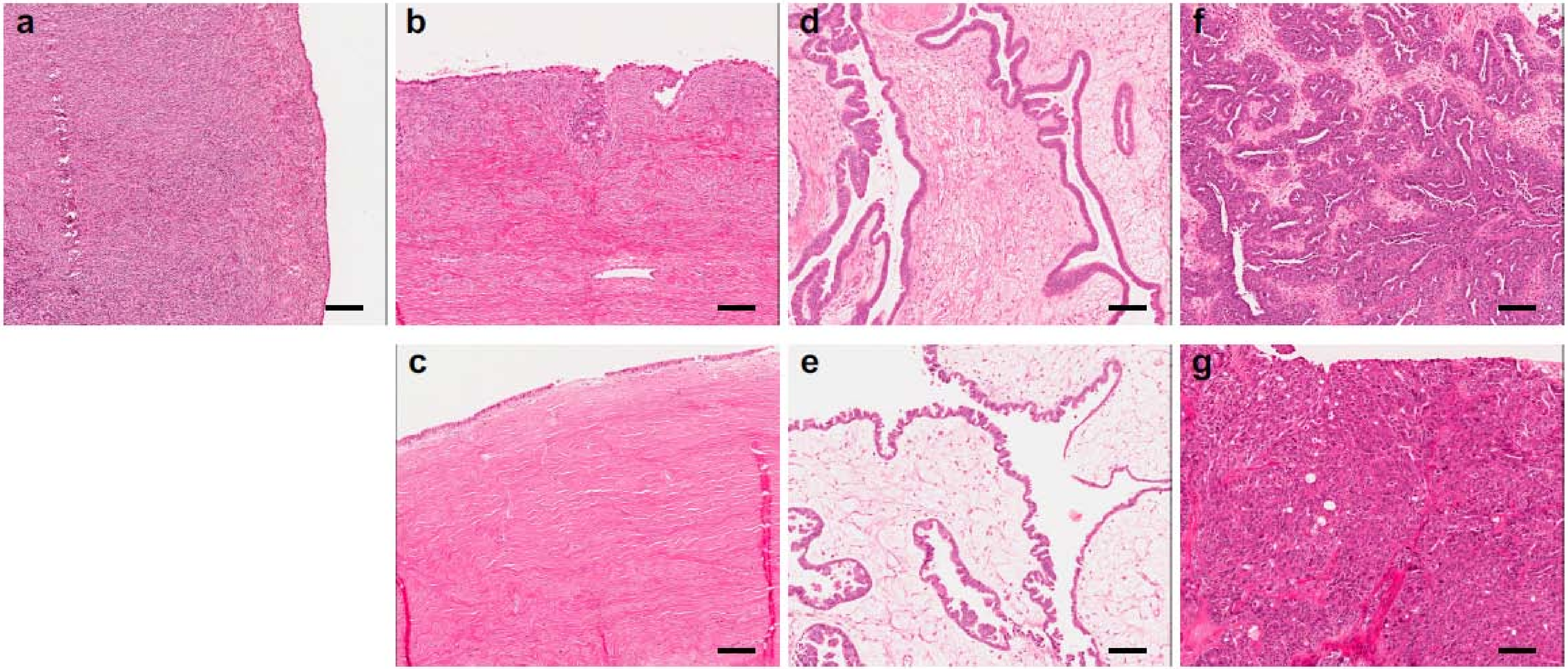

Additional file 6. Representative images of tumor tissues used in the study. H+E staining of representative tissues that were included as part of the study. a, Tissue number 44360 - normal ovary; b, Tissue number 44324 - benign tumor; c, Tissue number 44176 - benign tumor; d, Tissue number 44262 - LMP tumor; e, Tissue number 44047 - LMP tumor; f, Tissue number 44242 - invasive tumor; g, Tissue number 44306 - invasive tumor. Magnification - 200 x. Scale bar = 100 μm.
