# Supplementary material for: Expression profiling identifies genes involved in neoplastic transformation of serous ovarian cancer
Source: BMC Cancer. 2009 Oct 23;9:378. doi: 10.1186/1471-2407-9-378 (PMC2770078; doi:10.1186/1471-2407-9-378)
Supplement: Additional file 7 — WNT pathway and related molecules expression profiling data. Expression profiling data of all WNT pathway and related molecules present on the microarrays. [file 1471-2407-9-378-S7.PDF]

Additional file 7 – WNT pathway and related molecules expression profiling data

										Ratios		
**GenBank**	**Common**	**Normal Av**	**Normal SD**	**Benign Av**	**Benign SD**	**LMP Av**	**LMP SD**	**Invasive Av**	**Invasive SD**	**Invasive vs Normal**	**Invasive vs Benign**	**Invasive vs LMP**
AF038181	*APC*	0.79	0.59	0.73	0.44	0.90	0.21	1.03	0.22	1.30	1.41	1.14
NM_000038	*APC*	0.30	0.44	0.72	0.36	0.68	0.58	0.80	0.61	2.64	1.10	1.17
NM_005883	*APCL*	1.13	0.19	1.20	0.18	1.05	0.07	0.88	0.29	0.78	0.73	0.84
NM_001664	*ARHA*	0.89	0.60	0.67	0.32	1.01	0.32	1.08	0.43	1.21	1.62	1.07
NM_005172	*ATOH1*	1.03	0.22	0.78	0.31	0.91	0.32	1.08	0.37	1.05	1.37	1.18
AF009674	*AXIN1*	1.01	0.16	1.03	0.17	0.92	0.19	1.03	0.28	1.02	1.01	1.12
NM_004655	*AXIN2*	1.04	0.19	1.09	0.18	1.00	0.09	0.96	0.19	0.92	0.88	0.96
NM_033027	*AXUD1*	0.90	0.17	1.02	0.18	1.29	0.51	1.12	0.34	1.24	1.10	0.87
NM_001168	*BIRC5*	0.77	0.14	1.00	0.27	0.92	0.16	1.04	0.18	1.36	1.05	1.14
NM_001202	*BMP4*	1.04	0.17	0.89	0.17	0.98	0.12	1.03	0.20	0.99	1.16	1.05
NM_033637	*BTRC*	1.20	0.59	1.45	0.21	1.34	0.35	0.94	0.31	0.78	0.65	0.70
NM_031433	*C1QTNF5*	0.97	0.24	1.08	0.21	0.86	0.43	0.93	0.34	0.96	0.87	1.08
NM_006072	*CCL26*	1.12	0.16	1.02	0.19	0.86	0.44	0.99	0.34	0.89	0.97	1.16
NM_053056	*CCND1*	0.61	0.37	0.73	0.17	1.60	0.61	1.20	0.64	1.96	1.64	0.75
NM_001759	*CCND2*	0.68	0.47	1.15	0.56	1.10	0.39	0.91	0.48	1.34	0.79	0.83
NM_001760	*CCND3*	0.65	0.46	1.02	0.13	1.08	0.09	1.08	0.30	1.66	1.06	1.00
AJ251595	*CD44*	0.29	0.26	0.61	0.62	0.72	0.56	1.02	0.66	3.48	1.67	1.41
AF101051	*CLDN1*	0.64	0.46	0.83	0.27	1.36	0.42	1.00	0.39	1.57	1.21	0.73
NM_004380	*CREBBP*	0.75	0.55	1.01	0.21	0.92	0.13	1.12	0.42	1.50	1.11	1.22
NM_001892	*CSNK1A1*	0.76	0.15	0.79	0.10	1.10	0.13	1.07	0.19	1.41	1.35	0.97
NM_001893	*CSNK1D*	0.80	0.24	1.05	0.13	1.13	0.19	0.98	0.26	1.23	0.93	0.87
NM_001894	*CSNK1E*	1.02	0.11	1.20	0.27	1.03	0.32	1.11	0.33	1.08	0.92	1.07

Additional file 7 – WNT pathway and related molecules expression profiling data (cont'd)

GenBank	Common	Normal Av	Normal SD	Benign Av	Benign SD	LMP Av	LMP SD	Invasive Av	Invasive SD	Ratios		
										Invasive vs Normal	Invasive vs Benign	Invasive vs LMP
NM_001895	*CSNK2A1*	0.69	0.14	0.70	0.36	0.95	0.34	1.17	0.67	1.70	1.67	1.23
NM_001896	*CSNK2A2*	0.86	0.11	1.17	0.20	0.92	0.09	1.05	0.42	1.21	0.89	1.13
NM_001328	*CTBP1*	0.99	0.25	1.24	0.27	0.85	0.07	1.07	0.21	1.09	0.86	1.26
AL137653	*CTBP1*	0.16	0.32	0.35	0.62	0.14	0.37	0.20	0.41	1.22	0.56	1.42
NM_001903	*CTNNA1*	1.04	0.06	0.88	0.11	1.28	0.19	0.98	0.28	0.94	1.11	0.77
NM_004389	*CTNNA2*	0.97	0.11	0.96	0.10	0.97	0.17	0.99	0.27	1.03	1.03	1.03
NM_003798	*CTNNAL1*	1.85	0.62	1.61	0.41	1.09	0.29	0.82	0.35	0.44	0.51	0.76
Z37994	*CTNNAP1*	1.10	0.10	0.92	0.14	1.23	0.27	0.98	0.19	0.89	1.07	0.80
AF130085	*CTNNB1*	1.03	0.18	0.93	0.14	0.94	0.23	1.03	0.39	1.00	1.11	1.10
NM_001904	*CTNNB1*	1.05	0.11	1.16	0.11	1.01	0.08	0.94	0.11	0.90	0.81	0.93
NM_020248	*CTNNBIP1*	0.89	0.51	0.98	0.26	1.18	0.31	0.93	0.31	1.04	0.94	0.79
NM_030877	*CTNNBL1*	0.92	0.20	0.81	0.37	0.68	0.37	1.18	0.64	1.28	1.45	1.74
NM_001331	*CTNND1*	0.17	0.33	0.26	0.48	0.96	0.80	0.58	0.71	3.51	2.26	0.61
NM_001332	*CTNND2*	1.30	0.65	1.31	0.44	1.15	0.41	1.11	0.50	0.85	0.84	0.96
NM_016651	*DACT1*	1.73	0.89	1.78	0.64	1.01	0.12	0.94	0.40	0.54	0.53	0.93
NM_006848	*DIPA*	1.65	0.89	0.88	0.46	0.94	0.47	0.88	0.44	0.53	0.99	0.93
NM_012242	*DKK1*	0.92	0.88	1.57	1.78	0.46	0.38	1.33	0.95	1.45	0.85	2.88
NM_014421	*DKK2*	0.92	0.12	0.88	0.37	0.54	0.58	0.97	0.46	1.05	1.11	1.80
NM_015882	*DKK3*	1.08	0.82	0.67	0.68	1.56	2.83	1.10	1.97	1.02	1.64	0.71
NM_013253	*DKK3*	1.21	0.21	1.26	0.66	1.05	0.30	0.96	0.20	0.79	0.76	0.92
NM_014420	*DKK4*	1.29	0.21	1.29	0.37	1.06	0.20	0.99	0.34	0.76	0.77	0.93
NM_014419	*DKKL1-pending*	1.01	0.15	1.01	0.11	0.91	0.13	1.02	0.19	1.01	1.01	1.12
AL121723	*DKKL2*	0.99	0.01	1.09	0.18	0.94	0.24	1.01	0.22	1.03	0.93	1.08

Additional file 7 – WNT pathway and related molecules expression profiling data (cont'd)

GenBank	Common	Normal Av	Normal SD	Benign Av	Benign SD	LMP Av	LMP SD	Invasive Av	Invasive SD	Ratios		
										Invasive vs Normal	Invasive vs Benign	Invasive vs LMP
NM_004421	*DVL1*	0.96	0.29	0.91	0.23	1.01	0.24	1.05	0.42	1.09	1.15	1.04
NM_004422	*DVL2*	1.10	0.12	1.05	0.14	0.79	0.45	0.87	0.30	0.79	0.83	1.10
NM_004423	*DVL3*	1.09	0.11	1.15	0.18	0.83	0.11	1.05	0.21	0.97	0.92	1.26
NM_004429	*EFNB1*	0.73	0.22	0.98	0.11	1.16	0.24	1.10	0.25	1.50	1.12	0.95
NM_006209	*ENPP2*	0.89	0.13	1.19	0.54	0.99	0.18	1.06	0.31	1.18	0.89	1.06
NM_012164	*FBXW2*	3.38	4.94	1.03	0.21	0.78	0.47	0.85	0.51	0.25	0.82	1.08
AK054580	*FBXW2*	1.14	0.38	1.33	0.66	0.92	0.27	1.02	0.25	0.90	0.77	1.11
NM_003862	*FGF18*	0.93	0.27	0.92	0.46	1.45	0.69	1.02	0.39	1.10	1.12	0.70
NM_005252	*FOS*	3.05	2.21	2.49	1.56	1.09	0.90	1.31	1.47	0.43	0.53	1.20
NM_006732	*FOSB*	1.76	1.23	1.21	0.52	1.42	0.94	0.99	0.55	0.57	0.82	0.70
NM_005438	*FOSL1*	1.27	0.01	1.03	0.13	1.00	0.10	0.99	0.11	0.78	0.97	1.00
AK055579	*FOSL2*	1.67	2.50	0.84	0.69	1.14	0.95	1.51	1.52	0.90	1.81	1.33
NM_003593	*FOXN1*	1.04	0.20	0.93	0.18	1.00	0.13	1.02	0.21	0.98	1.09	1.02
NM_005479	*FRAT1*	1.23	0.04	1.01	0.20	1.15	0.38	1.07	0.28	0.87	1.06	0.93
NM_001463	*FRZB*	2.92	2.21	2.71	2.40	1.71	1.97	1.48	1.92	0.51	0.55	0.86
NM_006350	*FST*	1.12	0.25	1.10	0.31	1.06	0.32	1.01	0.27	0.90	0.92	0.95
NM_003505	*FZD1*	1.15	0.56	1.64	0.52	0.46	0.77	0.71	0.66	0.62	0.43	1.55
NM_007197	*FZD10*	0.91	0.43	1.24	0.86	1.15	0.58	1.36	2.06	1.48	1.10	1.18
NM_001466	*FZD2*	0.85	0.12	1.56	0.56	1.06	0.55	1.20	0.52	1.41	0.77	1.14
NM_017412	*FZD3*	1.10	1.20	0.96	0.26	1.23	0.88	1.17	0.56	1.06	1.21	0.95
NM_012193	*FZD4*	3.14	4.09	0.17	0.44	0.60	0.54	0.98	0.84	0.31	5.79	1.63
NM_003468	*FZD5*	0.76	0.51	0.89	0.62	0.71	0.49	0.90	0.34	1.19	1.02	1.28
NM_003506	*FZD6*	0.68	0.48	0.96	0.18	1.17	0.18	0.97	0.45	1.42	1.01	0.83

Additional file 7 – WNT pathway and related molecules expression profiling data (cont'd)

GenBank	Common	Normal Av	Normal SD	Benign Av	Benign SD	LMP Av	LMP SD	Invasive Av	Invasive SD	Ratios		
										Invasive vs Normal	Invasive vs Benign	Invasive vs LMP
NM_003507	*FZD7*	1.22	0.31	1.30	0.59	0.89	0.14	1.02	0.36	0.84	0.78	1.15
NM_031866	*FZD8*	1.27	0.13	1.11	0.20	0.96	0.10	1.05	0.19	0.83	0.95	1.10
NM_003508	*FZD9*	1.07	0.13	1.10	0.32	0.97	0.15	1.01	0.17	0.94	0.92	1.04
NM_002093	*GSK3B*	0.80	0.15	0.79	0.26	0.99	0.17	1.04	0.26	1.30	1.32	1.05
NM_004964	*HDAC1*	1.07	0.05	0.94	0.06	1.07	0.19	1.01	0.35	0.94	1.07	0.94
NM_025212	*IDAX*	1.14	0.31	1.00	0.08	0.99	0.22	0.96	0.26	0.85	0.96	0.98
NM_002228	*JUN*	1.50	0.55	1.22	0.39	0.84	0.26	1.09	0.55	0.73	0.90	1.30
NM_002229	*JUNB*	2.24	1.13	2.21	1.27	0.80	0.29	1.07	0.67	0.48	0.48	1.34
NM_005354	*JUND*	9.24	14.11	1.38	0.80	0.85	0.16	1.07	0.42	0.12	0.78	1.26
M28219	*LDLR*	0.62	0.75	1.38	1.58	1.09	1.72	1.23	1.30	1.99	0.89	1.13
NM_000527	*LDLR*	1.32	0.49	1.00	0.29	1.33	0.69	0.87	0.48	0.66	0.87	0.65
AF288571	*LEF1*	0.51	0.45	0.84	0.43	1.50	0.49	1.01	0.70	1.96	1.20	0.67
NM_005359	*MADH4*	1.25	0.34	1.24	0.40	1.07	0.26	0.90	0.36	0.72	0.73	0.84
NM_003188	*MAP3K7*	0.50	0.58	0.63	0.88	0.39	0.68	2.50	7.02	5.01	3.95	6.35
AY007145	*MAP3K7IP1*	1.10	0.04	0.98	0.16	0.98	0.23	1.01	0.20	0.92	1.03	1.03
NM_006116	*MAP3K7IP1*	1.18	0.30	1.06	0.27	0.94	0.11	1.00	0.19	0.85	0.95	1.07
NM_002753	*MAPK10*	1.65	0.47	1.35	0.40	0.69	0.46	0.90	0.70	0.55	0.66	1.30
NM_002752	*MAPK9*	1.10	0.06	0.86	0.14	0.97	0.22	1.03	0.17	0.94	1.20	1.06
NM_000245	*MET*	0.75	0.84	0.84	0.16	1.67	0.70	1.19	0.60	1.60	1.42	0.71
NM_002421	*MMP1*	0.85	0.69	0.98	0.45	0.92	0.25	1.33	0.91	1.56	1.35	1.45
NM_002425	*MMP10*	0.90	0.68	0.78	0.39	1.65	1.47	0.81	0.65	0.90	1.04	0.49
NM_005940	*MMP11*	0.87	0.37	0.80	0.69	0.81	0.25	2.83	5.32	3.24	3.52	3.49

Additional file 7 – WNT pathway and related molecules expression profiling data (cont'd)

										Ratios		
**GenBank**	**Common**	**Normal Av**	**Normal SD**	**Benign Av**	**Benign SD**	**LMP Av**	**LMP SD**	**Invasive Av**	**Invasive SD**	**Invasive vs Normal**	**Invasive vs Benign**	**Invasive vs LMP**
NM_002426	*MMP12*	0.95	0.33	0.87	0.21	1.04	0.32	1.58	2.86	1.66	1.81	1.53
NM_002427	*MMP13*	1.24	0.22	0.89	0.40	1.05	0.15	0.90	0.25	0.73	1.02	0.86
NM_004995	*MMP14*	0.97	0.73	1.12	0.34	0.92	0.10	1.16	0.60	1.20	1.04	1.27
NM_002428	*MMP15*	0.89	0.18	1.02	0.62	0.88	0.32	1.13	0.52	1.26	1.11	1.28
NM_005941	*MMP16*	1.12	0.25	1.48	0.71	1.00	0.21	1.51	1.16	1.35	1.02	1.52
NM_016155	*MMP17*	0.64	0.43	1.14	0.63	0.96	0.18	1.27	0.70	1.99	1.12	1.32
NM_022790	*MMP19*	0.90	0.82	1.20	0.23	1.04	0.25	0.94	0.32	1.05	0.78	0.91
NM_004530	*MMP2*	1.06	0.83	1.16	0.63	0.87	0.44	1.29	0.88	1.21	1.12	1.47
NM_004771	*MMP20*	1.38	1.05	1.11	1.47	1.46	0.78	1.03	1.01	0.75	0.93	0.70
NM_006690	*MMP24*	0.80	0.07	0.94	0.09	0.87	0.19	1.09	0.24	1.35	1.16	1.26
NM_022468	*MMP25*	0.66	0.46	0.85	0.39	0.94	0.19	1.02	0.31	1.55	1.20	1.09
NM_004142	*MMP25*	1.21	0.12	0.94	0.17	1.17	0.38	1.11	0.57	0.92	1.18	0.95
NM_021801	*MMP26*	1.19	0.55	1.16	0.37	1.27	0.54	1.02	0.23	0.86	0.88	0.80
NM_022122	*MMP27*	0.79	0.82	2.20	2.83	0.71	0.53	0.95	0.69	1.20	0.43	1.34
NM_032950	*MMP28*	0.94	0.27	0.96	0.64	0.72	0.34	0.97	0.60	1.03	1.01	1.34
NM_002422	*MMP3*	0.21	0.41	0.87	1.04	0.35	0.55	0.62	0.66	2.96	0.71	1.75
NM_002423	*MMP7*	0.87	0.20	0.97	0.49	0.81	0.67	0.93	0.55	1.06	0.96	1.14
NM_002424	*MMP8*	0.00	0.01	0.00	0.00	0.00	0.00	29.88	97.99	11950.29	20913.00	20913.00
NM_004994	*MMP9*	1.06	0.49	1.23	0.99	1.00	0.35	2.30	5.40	2.17	1.87	2.29
NM_002467	*MYC*	1.18	0.28	1.18	0.39	1.15	0.24	1.00	0.51	0.85	0.85	0.87
BC002712	*MYCN*	0.00	0.01	0.36	0.61	1.70	2.65	0.62	1.57	246.50	1.72	0.36
NM_016231	*NLK*	1.07	0.16	1.00	0.15	0.99	0.47	0.96	0.26	0.89	0.96	0.96
NM_005010	*NRCAM*	0.12	0.15	0.16	0.42	0.84	1.19	1.02	1.47	8.15	6.27	1.21

Additional file 7 – WNT pathway and related molecules expression profiling data (cont'd)

GenBank	Common	Normal Av	Normal SD	Benign Av	Benign SD	LMP Av	LMP SD	Invasive Av	Invasive SD	Ratios		
										**Invasive vs Normal**	**Invasive vs Benign**	**Invasive vs LMP**
NM_000430	*PAFAH1B1*	1.09	0.16	1.69	0.51	1.05	0.20	0.95	0.37	0.88	0.56	0.91
NM_002658	*PLAU*	1.04	0.47	0.85	0.26	0.77	0.41	1.71	1.95	1.64	2.02	2.22
NM_002659	*PLAUR*	0.73	0.57	0.62	0.63	0.80	0.77	0.98	0.82	1.34	1.59	1.23
NM_006238	*PPARD*	0.72	0.49	1.14	0.16	0.90	0.10	0.99	0.18	1.38	0.87	1.11
NM_002715	*PPP2CA*	0.72	0.56	0.79	0.34	1.01	0.22	0.90	0.48	1.24	1.14	0.88
NM_004156	*PPP2CB*	1.23	0.35	1.57	0.36	1.05	0.26	0.98	0.32	0.80	0.63	0.94
NM_002719	*PPP2R5C*	0.85	0.59	1.05	0.49	1.14	0.62	1.23	0.84	1.45	1.17	1.07
NM_006246	*PPP2R5E*	0.82	0.29	0.93	0.25	1.08	0.20	1.05	0.22	1.28	1.12	0.97
AF035594	*PRKCA*	1.51	1.42	0.87	0.20	1.15	0.42	1.09	0.39	0.72	1.25	0.95
NM_002737	*PRKCA*	2.02	1.35	1.29	0.41	1.04	0.80	0.86	0.30	0.43	0.67	0.82
NM_002738	*PRKCB1*	0.66	0.54	0.88	0.21	0.92	0.14	1.08	0.20	1.63	1.23	1.17
AK057555	*PRKCB1*	1.01	0.13	1.28	0.41	1.01	0.10	0.91	0.39	0.89	0.71	0.89
NM_006254	*PRKCD*	0.87	0.19	1.00	0.19	1.13	0.21	1.01	0.28	1.16	1.01	0.89
NM_005400	*PRKCE*	0.81	0.09	0.81	0.37	1.06	0.09	1.05	0.19	1.30	1.29	0.99
NM_002739	*PRKCG*	0.11	0.22	0.00	0.00	0.87	1.47	0.65	0.99	5.81	454.58	0.75
NM_024064	*PRKCH*	0.86	0.25	0.84	0.37	1.16	0.30	1.01	0.38	1.18	1.20	0.87
NM_006255	*PRKCH*	0.90	0.12	0.95	0.23	1.12	0.22	1.03	0.26	1.15	1.09	0.93
NM_002740	*PRKCI*	0.36	0.71	0.61	0.92	0.21	0.26	0.73	0.79	2.07	1.21	3.57
NM_002742	*PRKCM*	1.06	0.21	0.99	0.27	1.08	0.12	0.99	0.23	0.94	1.00	0.92
NM_005813	*PRKCN*	0.92	0.39	0.96	0.31	0.93	0.34	1.31	0.92	1.43	1.36	1.41
NM_006257	*PRKCQ*	1.30	0.68	1.11	0.13	1.04	0.14	1.03	0.31	0.79	0.93	0.99
NM_002744	*PRKCZ*	0.58	0.51	0.69	0.92	0.90	0.55	1.00	0.57	1.73	1.45	1.11
AK054993	*RAC1*	1.18	0.32	0.94	0.26	0.96	0.52	0.97	0.27	0.82	1.04	1.02

Additional file 7 – WNT pathway and related molecules expression profiling data (cont'd)

										Ratios		
**GenBank**	**Common**	**Normal Av**	**Normal SD**	**Benign Av**	**Benign SD**	**LMP Av**	**LMP SD**	**Invasive Av**	**Invasive SD**	**Invasive vs Normal**	**Invasive vs Benign**	**Invasive vs LMP**
NM_003012	*SFRP1*	1.10	0.10	1.28	0.40	1.01	0.17	0.83	0.32	0.75	0.65	0.82
AF311912	*SFRP2*	0.84	0.10	0.87	0.13	1.01	0.35	1.32	0.55	1.58	1.51	1.30
NM_003014	*SFRP4*	1.58	1.20	1.94	1.81	0.81	0.45	1.20	0.92	0.76	0.62	1.48
NM_003015	*SFRP5*	1.22	0.14	1.04	0.18	0.97	0.07	0.98	0.18	0.81	0.94	1.01
NM_005631	*SMO*	1.11	0.13	0.99	0.23	1.16	0.34	1.08	0.37	0.98	1.09	0.94
M57732	*TCF1*	1.09	0.10	1.16	0.15	0.86	0.12	1.09	0.50	1.00	0.94	1.27
NM_003202	*TCF7*	0.79	0.46	1.42	0.58	1.01	0.25	1.04	0.34	1.31	0.74	1.03
NM_031283	*TCF7L1*	1.21	0.34	1.08	0.33	0.82	0.33	1.07	0.39	0.89	1.00	1.31
NM_005077	*TLE1*	1.22	0.85	1.34	0.24	1.12	0.28	0.96	0.29	0.79	0.72	0.86
NM_003260	*TLE2*	0.93	0.14	0.97	0.07	0.84	0.38	1.00	0.36	1.07	1.03	1.19
AB046767	*TLE3*	1.06	0.09	0.82	0.41	0.96	0.14	0.91	0.31	0.86	1.12	0.95
AK057236	*TLE4*	1.77	1.24	1.75	1.10	1.21	0.50	0.88	0.51	0.50	0.50	0.72
AF022375	*VEGF*	0.89	0.12	1.00	0.25	0.95	0.25	1.02	0.21	1.15	1.02	1.08
AK056914	*VEGF*	0.80	0.56	1.40	0.66	0.86	0.19	1.28	0.77	1.61	0.92	1.49
NM_003377	*VEGFB*	1.01	0.10	1.05	0.19	0.88	0.17	1.02	0.28	1.01	0.97	1.15
NM_005429	*VEGFC*	0.77	0.51	0.98	0.20	1.02	0.17	0.98	0.30	1.27	1.00	0.96
NM_013266	*VR22*	1.42	0.71	3.12	3.27	1.02	1.36	0.96	0.78	0.67	0.31	0.94
NM_007191	*WIF1*	0.93	0.06	1.01	0.36	0.93	0.58	1.23	0.50	1.32	1.21	1.32
AK027294	*WISP1*	0.49	0.97	0.00	0.00	0.50	0.78	0.44	0.68	0.89	305.00	0.87
NM_003882	*WISP1*	1.06	0.13	1.52	1.22	1.12	0.29	0.98	0.28	0.93	0.65	0.88
NM_003881	*WISP2*	1.51	0.48	1.16	0.13	0.92	0.27	0.98	0.21	0.65	0.84	1.06
NM_003880	*WISP3*	0.70	0.56	1.24	0.38	1.13	0.32	0.92	0.34	1.32	0.74	0.81
NM_005430	*WNT1*	1.02	0.16	1.24	0.22	1.03	0.23	0.94	0.28	0.92	0.76	0.91

Additional file 7 – WNT pathway and related molecules expression profiling data (cont'd)

GenBank	Common	Normal Av	Normal SD	Benign Av	Benign SD	LMP Av	LMP SD	Invasive Av	Invasive SD	Ratios: Invasive vs Normal	Ratios: Invasive vs Benign	Ratios: Invasive vs LMP
NM_003391	*WNT2*	0.94	0.31	1.17	0.20	1.83	0.44	0.94	0.24	1.00	0.80	0.52
NM_024494	*WNT2B*	1.46	0.74	1.10	0.68	1.03	0.37	0.90	0.38	0.61	0.82	0.87
NM_030753	*WNT3*	0.00	0.01	0.08	0.22	2.28	5.64	1.32	2.64	529.69	15.90	0.58
NM_033131	*WNT3A*	1.07	0.21	1.20	0.35	1.01	0.10	0.95	0.23	0.89	0.79	0.94
AB062766	*WNT4*	0.97	0.13	1.10	0.24	0.94	0.15	0.98	0.20	1.01	0.89	1.04
NM_030761	*WNT4*	1.00	0.08	1.14	0.16	1.10	0.26	0.98	0.20	0.99	0.86	0.89
NM_003392	*WNT5A*	0.91	0.62	1.36	0.44	1.10	0.33	0.79	0.35	0.87	0.58	0.72
NM_032642	*WNT5B*	1.13	0.22	0.91	0.16	0.96	0.23	1.05	0.19	0.93	1.15	1.09
NM_006522	*WNT6*	0.87	0.57	1.16	0.16	0.93	0.11	1.05	0.19	1.21	0.91	1.13
NM_004625	*WNT7A*	0.61	0.19	0.60	0.13	0.71	0.28	1.26	0.47	2.05	2.09	1.77
NM_031933	*WNT8A*	0.52	0.69	0.09	0.23	2.08	3.51	2.02	4.72	3.85	22.95	0.97
NM_003393	*WNT8B*	1.27	0.40	1.29	0.33	1.55	1.44	1.03	0.43	0.81	0.80	0.66
NM_003395	*WNT9A*	1.14	0.13	1.06	0.20	1.09	0.34	0.99	0.31	0.87	0.94	0.91
NM_003396	*WNT9B*	1.00	0.12	0.92	0.49	0.99	0.35	1.15	0.71	1.15	1.25	1.16
NM_025216	*WNT10A*	1.23	0.17	1.19	0.28	1.02	0.19	0.95	0.20	0.78	0.80	0.93
NM_003394	*WNT10B*	0.54	0.62	0.65	0.63	1.04	1.17	0.73	0.69	1.35	1.12	0.70
NM_004626	*WNT11*	0.91	0.19	1.00	0.20	0.96	0.10	0.97	0.27	1.06	0.97	1.00
NM_057168	*WNT16*	1.01	0.38	0.74	0.36	1.22	0.38	1.02	0.41	1.01	1.39	0.84
